# Leadership and Path Characteristics during Walks Are Linked to Dominance Order and Individual Traits in Dogs

**DOI:** 10.1371/journal.pcbi.1003446

**Published:** 2014-01-23

**Authors:** Zsuzsa Ákos, Róbert Beck, Máté Nagy, Tamás Vicsek, Enikő Kubinyi

**Affiliations:** 1 Department of Biological Physics, Eötvös University, Budapest, Hungary; 2 Statistical and Biological Physics Research Group of the Hungarian Academy of Sciences, Budapest, Hungary; 3 Department of Zoology, University of Oxford, Oxford, United Kingdom; 4 MTA-ELTE Comparative Ethological Research Group, Budapest, Hungary; Imperial College London, United Kingdom

## Abstract

Movement interactions and the underlying social structure in groups have relevance across many social-living species. Collective motion of groups could be based on an “egalitarian” decision system, but in practice it is often influenced by underlying social network structures and by individual characteristics. We investigated whether dominance rank and personality traits are linked to leader and follower roles during joint motion of family dogs. We obtained high-resolution spatio-temporal GPS trajectory data (823,148 data points) from six dogs belonging to the same household and their owner during 14 30–40 min unleashed walks. We identified several features of the dogs' paths (e.g., running speed or distance from the owner) which are characteristic of a given dog. A directional correlation analysis quantifies interactions between pairs of dogs that run loops jointly. We found that dogs play the role of the leader about 50–85% of the time, i.e. the leader and follower roles in a given pair are dynamically interchangable. However, on a longer timescale tendencies to lead differ consistently. The network constructed from these loose leader–follower relations is hierarchical, and the dogs' positions in the network correlates with the age, dominance rank, trainability, controllability, and aggression measures derived from personality questionnaires. We demonstrated the possibility of determining dominance rank and personality traits of an individual based only on its logged movement data. The collective motion of dogs is influenced by underlying social network structures and by characteristics such as personality differences. Our findings could pave the way for automated animal personality and human social interaction measurements.

## Author Summary

How does a group of family dogs decide the direction of their collective movements? Is there a leader, or is decision-making based on an egalitarian system? Is leadership related to social dominance status? We collected GPS trajectory data from an owner and her six dogs during several walks. We found that dogs adjusted their trajectories to that of the owner, that they periodically run away, then turn back and return to her in a loop. Tracks have unique features characterising individual dogs. Leading roles among the dogs are frequently interchanged, but leadership is consistent on a long timescale. Decisions about running away and turning back to the owner are not based on an egalitarian system; instead, leader dogs exert a disproportionate influence on the movement of the group. Leadership during walks is related to the dominance rank assessed in everyday agonistic situations; thus, the collective motion of a dog group is influenced by the underlying hierarchical social network. Leader/dominant dogs have a unique personality: they are more trainable, controllable, and aggressive, additionally they are older than follower/subordinate dogs. Dogs are an ideal model for understanding human social behaviour. Therefore, we address the possibility of conducting similar studies in humans, e.g. walking with children and detecting interactions between individuals.

## Introduction

Groups that are not able to coordinate their actions and cannot reach a consensus on important events, such as where to go, will destabilise, and individuals will lose the benefits associated with being part of a group [1], [2]. Decision-making usually involves some form of leadership, i.e. ‘the initiation of new directions of locomotion by one or more individuals, which are then readily followed by other group members’ ([3] p83).

Several factors may give rise to the emergence of leadership. In some species or populations, leaders are socially dominant individuals (consistent winners of agonistic interactions [4]) and have more power to enforce their will [5]. For example, in rhesus macaques (*Macaca mulatta*) the decision to move is the result of the actions of dominant and old females [6]. Similarly, dominant beef cows (*Bos taurus*) have the most influence on where the herd moves. They go where they wish while subordinates either avoid or follow them [7].

Leaders could appear in species or populations without any dominant individuals, or independently from social dominance. Leaders may have the highest physiological need to impose their choice of action [1], [3], [8]–[10], or they may possess special information or skill [11], [12].

Finally, an individual of a personality type that is more inclined to lead or does not prefer following others may also initiate collective movements [13], [14]. For example, leadership is associated with boldness in sticklebacks (*Gasterosteus aculeatus*) [15], [16]. The investigation of the relationship between leadership and personality might reveal which personality types occupy particular positions in the leadership network, and conversely, network metrics could identify potential personality traits.

With this study our aim was to reveal potential links between leadership in collective movements, motion patterns, social dominance, and personality traits in domestic dogs (*Canis familiaris*). It is often assumed that domestic dogs inherited complex behaviours from their wolf ancestors (*Canis lupus*). The typical wolf pack is a nuclear or extended family, where the dominant/breeding male initiates activities associated with foraging and travel [17]. However, family dog groups may consist of several unrelated individuals with multiple potential breeders. In large wolf packs with several breeders, leadership varies among packs, and dominance status has generally no direct bearing on leadership, but breeders tend to lead more often than non-breeders [18]. Similarly, leadership in Italian free-ranging dogs interchanged between a small number of old and high-ranking habitual leaders. Interestingly, affiliative relationships had more influence on leadership than agonistic interactions [19].

Family dogs are often kept in groups (for instance, 33% of owners in Germany [20] and 26% of owners in Australia [21] have 2 or more dogs), however interactions within freely moving dog groups and their relationship with social dominance are still unexplored. The capacity of dogs to form robust dominance hierarchies is highly debated [22], [23]. However, the reason for the inability to detect hierarchies might be due to methodological issues in certain cases, as instead of aggression patterns, submissive behaviours appear to be better indicators of dominance relationships in dogs [24].

To describe what characterises the collective movement of a group of dogs, and to investigate links between leadership, social dominance, personality [25], and characteristics of individual motion trajectories, we collected high-resolution spatio-temporal (1–2 m, 0.2 s) GPS trajectory data from a group of dogs and their owner during everyday walks. Directional choice dynamics and potential leading activity were assessed by quantitative methods inspired by statistical physics [26], [27]. Personality and dominance rank of the dogs were measured by questionnaires completed by the owner. Because the capacity to form dominance hierarchies is likely to vary from breed to breed [28], we chose a group that contains multiple individuals of the same breed, the Hungarian Vizsla. The studied group is composed of five Vizslas (with two dam-offspring pairs) and one small-sized, mixed-breed dog.

## Results

### Characteristics of the paths

**Figure 1:**
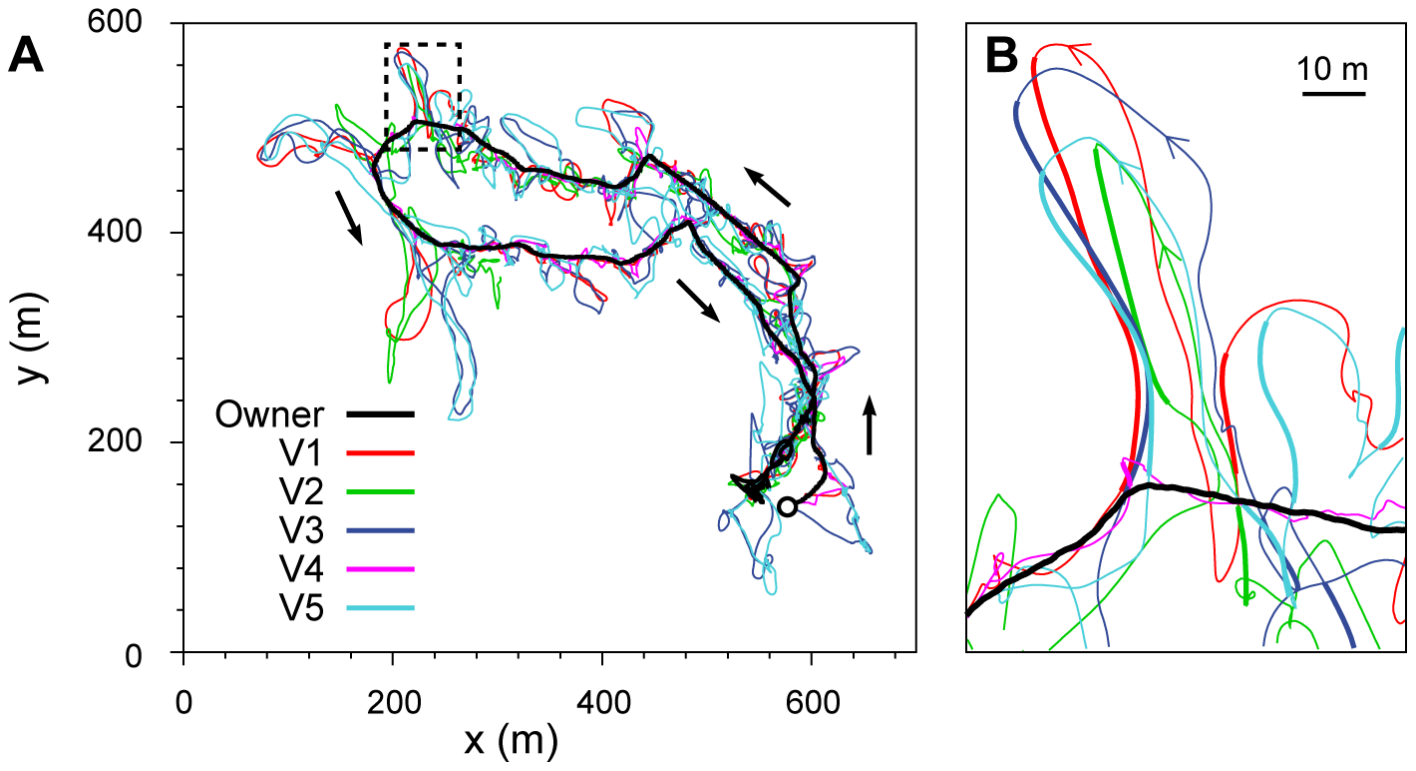
A typical walk of the group and the illustration of returning loops. (**A**) Highly detailed trajectories of the dogs (only Vizslas are shown) and of the owner during a 30-minute walk. Arrows indicate the direction of motion. (**B**) The dogs run in loops and return from time to time to the owner. Thickened segments of the tracks show when a dog's return to its owner was found by our automated method.

A general overview of the GPS-logged trajectories (see Figure 1 and Video S1: our animation showing a 3-minute-long part of a walk) shows that the dogs run away from the owner periodically, then turn back and return to her, in a loop. Figure S5 shows a typical trajectory of dog V1. It can also be seen that they prefer running these loops or a part of them with one or more group members (see details in the Data Analysis). Given that the dogs' speed was significantly higher than that of the owner (1.5–3.7 times), this motion pattern allows dogs to cover a greater distance than the owner while also keeping the group together. We calculated several simple characteristics of the trajectories and performed an analysis concerning the returning events (Table 1 and Text S1).

**Table 1:** Relevant variables describing the characteristics of dogs' paths and variables extracted from the returning event analysis for each subject (Vizslas; V1 to V5 and the mixed-breed dog; M).

Dogs	V1	V2	V3	V4	V5	M
**Preferred running speed (m/s)**	2.6±1.0	3.0±0.5	3.4±0.6	1.5±0.4	4.0±0.5	1.4±0.2
**Relative distance covered**	2.4±0.7	2.3±0.3	3.7±1.0	1.8±0.5	3.2±0.7	1.5±0.2
**Distance from the owner (m)**	10.3±4.2	16.9±4.0	20.2±5.3	9.0±2.4	23.3±6.5	13.7±4.4
**Distance from dogs (m)**	16.0±3.3	17.0±1.4	18.1±2.6	15.9±3.7	19.9±2.6	19.0±3.6
**Time period of the returns (s)**	52±47	52±47	40±37	75±74	52±49	108±94
**Loop length (m)**	16±16	20±14	20±17	12±10	24±21	22±20
**Far-from-owner ratio**	0.45±0.17	0.56±0.07	0.50±0.12	0.56±0.06	0.54±0.08	0.59±0.11

For each variable, averages over the walks and standard deviations between the walks are shown (Mean ± SD), except for time period of the returns and loop length, where the SD of all data is indicated. See more details in Text S1.

The preferred running speeds of the dogs, the relative distances covered, and the distances from the owner were unique and consistent characteristics of an individual dog's path, while other characteristics (e.g, distance from dogs) were less consistent and/or distinctive (for details see Text S1).

### Interactions

**Figure 2:**
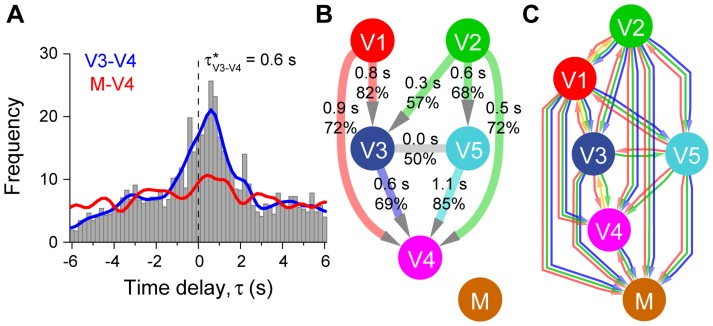
Directional correlations between tracks of dog pairs, the resulting leadership network and the results of the dominance questionnaire. (**A**) Directional correlation delay time (τ) values for a given pair (V3 and V4) when high correlation was found for a time window shown by the grey histogram, while the blue curve shows the function gained by Gaussian smoothing with σ = 0.3 s. The distribution shows a clear peak at τ* = 0.6 s. For a comparison, the red curve shows a directional correlation delay time function for another pair (M and V4), where no connection was found between the two dogs in the absence of a significant peak. (**B**) Summarised leadership network composed of the directional delay time values. Each directed link points from the individual that plays the role of the leader more often in the given relationship toward the follower. The grey link shows a strong connection between V3 and V5 with an evenly matched relationship (τ* = 0 s). The upper values on the edges indicate the mode of time delays in seconds and the lower values show the average portion that the leader of that pair was actually leading. Note that these modes are from wide distributions (as shown on panel A) with an average full width at half maximum of 3.7 s. The mixed-breed (M) is not connected to any Vizslas, and so is not part of the network. This network is used to calculate leading tendency, which is the number of followers that can be reached travelling through directed links. (**C**) Dominance network between the dogs derived from the dominance questionnaire [29]. Each directed edge points from the dominant individual toward the subordinate one. The colours represent the context when dominance is evident: red: barking, orange: licking the mouth, green: eating and blue: fighting (see more details in Text S1). The nodes were arranged in the vertical direction in such a way that more edges point downwards than upwards between all pairs.

To extract information about the interactions between group members, we used a directional correlation analysis [26] with a time window to quantify the fast, joint direction changes for all possible pairings of the dogs (Figure 2A; Table S1; for more details see Data Analysis and Text S1).

We detected frequent short-term interactions and leading tendency differences between dog pairs within the group. The leading and following roles between interacting pairs were often changed during walks and between walks. To check the robustness of the interactions, the directional delay times were calculated for the first 7 and the second 7 walks separately for all pairs. High correlation was found (two-tailed Pearson correlation: *r* = 0.635, *n* = 15, *p* = 0.011), i.e. significant differences in leading tendency were detected over longer timescales. Calculated from a Gaussian fit to the peak of the relevant distributions (Figure S8, Table S1) we found that dogs play the role of leader in a given pair about 50–85% of the time (57% to 85% when directed leader-follower relationships were found).

Based on the directional delay time values, we created a summarised leadership network (Figure 2B). In the network each directed link points from the individual, which played the role of the leader more often in that given relationship toward the follower. We used this network to calculate leading tendency, which is the number of followers that can be reached travelling through directed links.

We also calculated ‘active connections’, which shows the number of how many interactions a dog has (with the number of edges a dog is connected with in the network).

### Relationships between the trajectory variables, leading tendency, dominance ranks, and personality traits

**Figure 3:**
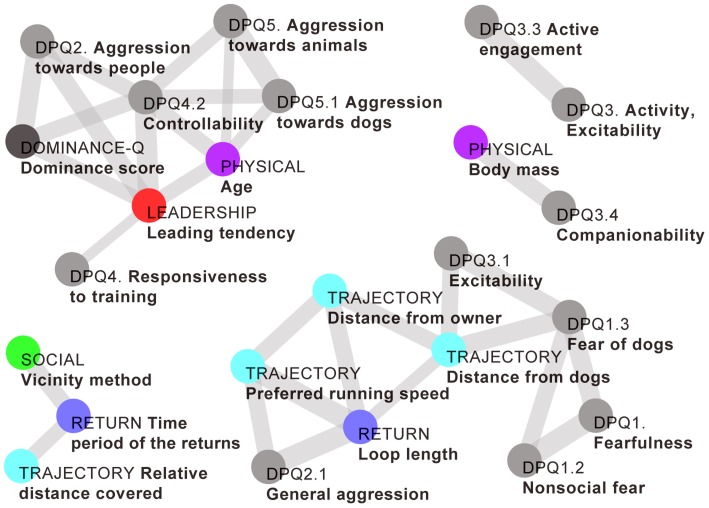
Significant correlations of variables calculated from trajectory data with the personality traits of the dogs measured by questionnaires. The figure shows the significant correlations (where *p*<0.05) between the variables (edge width indicating the strength of correlation) calculated for the Vizslas (*n* = 5). The first term of each name and the colouring of the nodes show the origin of each variable: DPQ: Dog Personality Questionnaire ([51]; gray), Dominance-Q: dominance questionnaire ([29]; dark gray); Physical: physical attributes of the dogs (purple); Trajectory: simple characteristics from the trajectories (cyan); Return: relevant characteristics of the returns to the owner (blue); Social: number of social connection to other dogs calculated from trajectories (green); Leadership: leadership hierarchy from directional correlation delays (red). Only those questionnaire variables are shown which had significant correlation with any variable of another type. All connections are shown between the variables presented on the plot.

Correlations between trajectory-based variables, leading tendency, personality traits (Jones, 2008, Table 2) and dominance rank (Pongrácz et al., 2008, Table 2) were calculated using two-tailed Pearson correlation for the Vizslas only (*n* = 5) (Figure 3) and also for all subjects (*n* = 6). We tested our data for normality using a Shapiro-Wilk test (p<0.05), and where a significant deviation from a normal distribution was found, we used Spearman correlations (indicated as *r*_S_).

**Table 2:** Demographic variables and factor scores of dogs.

Dogs	V1	V2	V3	V4	V5	M
**Breed**	Vizsla	Vizsla	Vizsla	Vizsla	Vizsla	Mix
**Sex**	female	female	male	female	female	female
**Neutered status**	intact	neutered	intact	intact	intact	intact
**Age at adopting (years)**	2	0	0	0.2	0	2
**Age at the end of measurement (years)**	6.5	7	1.5	1	4	4
**Weight (body mass) at the end of measurement (kg)**	28	26	26	26	25	10
**DPQ1. Fearfulness**	2.00	1.08	2.67	1.25	4.08	3.42
**DPQ2. Aggression towards people**	2.17	2.83	2.17	1.50	2.00	1.33
**DPQ3. Activity/Excitability**	4.67	5.00	5.00	5.17	5.17	4.67
**DPQ4. Responsiveness to training**	5.83	6.83	5.50	5.50	5.50	2.17
**DPQ5. Aggression towards animals**	5.33	5.33	4.33	3.44	5.22	4.11
**DOMINANCE Questionnaire**	10	14	10	2	8	1

(DPQ: Dog Personality Questionnaire).

Our main aim was to investigate whether the leadership we defined based on the motion patterns had any connection with the social dominance.We found that the leading tendencies calculated from the GPS data significantly correlated with the dominance ranks gained from the dominance questionnaire [29] (*r* = 0.92, *n* = 5, *p* = 0.026). To support this result, we performed a comparison with a randomisation using all possible permutations, and this correlation value proved to be significantly higher than it was for the randomised cases. For more details see Text S1 and Figures SI11–13.

To find more correlations in our dataset of trajectory variables and personality traits, all 300 possible pairings were analysed. Note that due to the large number of variable pairs and the small number of dogs involved in the study, none of the p-values remain significant after correction for multiple comparisons (Bonferroni, Sidak or Benjamini–Hochberg procedure). But the correlations mentioned here were all significantly higher than the corresponding values of the randomly permuted cases.

The distance from other dogs correlated with the fear of dogs facet (*r*_S_ = 0.92, *n* = 5, *p* = 0.028) and the excitability facet (*r*_S_ = 0.92, *n* = 5, *p* = 0.026). Dogs that, according to the owner, avoid other dogs and seek constant activity maintained a longer distance from their group mates during the walks.

The time period of the returns (the average time duration between returning events) was found to be inversely correlated with the controllability facet (*r* = −0.82, *n* = 6, *p* = 0.046), and the dominance rank measure (*r* = −0.84, *n* = 6, *p* = 0.036). Dominant dogs who were more responsive to training returned to the owner more often.

The far-from-owner ratio (the time ratio of being relatively far from the owner, for more details see Text S1) correlated negatively with companionability (*r* = −0.87, *n* = 6, *p* = 0.024). Dogs that, according to the owner, seek companionship from people also like staying in the owners' proximity.

The preferred running speed correlated with the general aggression facet of the aggression toward people factor (*r* = 0.95, *n* = 5, *p* = 0.015). More aggressive dogs ran faster during the walks.

In addition to being correlated with dominance rank (mentioned earlier), leading tendency was positively correlated with: age (*r* = 0.91, *n* = 5, *p* = 0.032), responsiveness to training (*r*_S_ = 0.92, *n* = 5, *p* = 0.028), controllability (*r* = 0.98, *n* = 5, *p* = 0.003), and aggression towards people (*r* = 0.95, *n* = 5, *p* = 0.013). These relations indicate that those dogs that have a tendency to take the leading role during walks are more aggressive and dominant, and they are also more controllable by the owner, based on the personality questionnaires (Figure 3).

## Discussion

By analyzing the GPS trajectories of freely moving dogs and their owner during walks, we found significant differences in simple path characteristics of the individual dogs. The preferred running speed of Vizslas ranged from 1.5 to 4.0 m/s (5.4–14.4 km/h), they covered a 1.8–3.7× longer distance than the owner during a walk, and the usual distances from the owner ranged from 16 to 20 m. These results might be useful for conservation managers in establishing areas where dog walking is prohibited [30] and may also help in designing parks, as dog-walking is a popular method for increasing human physical activity (for a review, see [31]).

A directional correlational analysis [26], [27] revealed leader-follower interactions between the group members. We detected a loose but consistent hierarchical leadership structure. Due to the dynamic nature of the pairwise interactions, role reversals did occur during walks and an individual took the role of the leader in a given pair in about 73% (ranging from 57% to 85%) of their interactions, where directed leader-follower relationships were found. This ratio is of similar magnitude to the case of wild wolf packs with several breeding individuals, where leaders led for 78% of the recorded time, ranging from 58% to 90% [18]. The role of initiating common actions is also frequently interchanged between guide dogs and the owner [32] and between dogs during play [33]. But over a longer timescale, differences in leading tendency remained consistent; thus decision-making during the collective motion was not based on an egalitarian system in our sample.

Although the existence of an overall dominance hierarchy in dogs is debated [23], and the Vizsla is a “peaceful” breed, which, compared to other breeds, rarely fights with conspecifics [34], we detected a dominance hierarchy via a questionnaire assessing agonistic and affiliative situations [29]. We found that dominance rank and leadership were strongly connected. Dogs who tend to win in everyday fighting situations, eat first, bark more or first, and receive more submissive displays from the others, and have more influence over the decisions made during collective motion.

The correlation between leadership and dominance is consistent with a trend in ‘despotic’ social mammals [5], but probably not characteristic in wolves with several breeding individuals [18]. In large wolf packs (with 7–23 individuals), breeding individuals lead during travels, independently from dominance status. But this situation is relatively rare, as the typical wolf pack is a nuclear or extended family, where the only breeding male leads the pack during travel [17]. Unlike wolves, the dog is a promiscuous species, and in a group, there is usually no single pair of breeders [22]. In our family dog group, the highest ranking dog (V2) was neutered, which may suggest that both leadership and dominance have little or no relationship with reproductive behaviour in family dogs, consistent with observations in feral dogs in India [35]–[37].

We also investigated the relationship between leadership and personality to reveal which personality types occupy particular positions in the leadership network. We found that leaders/dominants were more responsive to training, more controllable, and more aggressive than followers/subordinates. Other data also suggest that dominance cuts across different contexts and is correlated with boldness, extraversion, and exploratory tendencies in several taxa [38], and assertiveness in wolves [18], but reported links between personality and leadership are rare [14].

Age was a reliable indicator of leadership and dominance. Several studies have reported a positive correlation between age and dominance [39]. Age-related dominance might be due to greater fighting skills (e.g. [40]) or enhanced possibility of forming alliances with other individuals, among other factors [41]. If rank acquisition is learnt at an early age with regular reassessments of dominance, younger dogs may remain subordinate, long after initial body weight differences have disappeared. In our group, both dams were dominant over their adult offsprings, and each adult Vizsla dominated the juvenile Vizsla, which supports the hypothesis that the acceptance of subordinate status within a dog group is probably mediated by conditioning.

Not only leadership and dominance, but movement characteristics were also related to personality. Fearful and excitable dogs maintained a longer distance from other dogs. More controllable and dogs returned to the owner more often, while less companionable dogs spent more time far from the owner. Surprisingly, more aggressive dogs ran faster during the walks. As male dogs harvest more game than females in preindustrial societies [42], and experimental evidence on mice suggests that testosterone increases persistence of food searching in rodents [43], higher speed might be related to testosterone levels. Note, however, that even the most “aggressive” score was relatively low in our sample (2.67 out of the maximum 8).

Social organization and social structure vary among populations [44], and in the case of dogs, they vary among breeds and groups [45], thus group decision-making processes are expected to vary accordingly [46]. The main limitation of our study is the low sample size. Observing other groups and breeds may provide different results. For example, the hierarchical network of sled dogs which work as a team with a lead dog [47] is more robust than that of our sample. It would also be interesting to investigate what happens with the leadership network if the owner runs or rides a bike, and her speed is comparable to the dogs' speed.

To summarise, by using GPS devices we found that the leader and follower roles are dynamically interchanged during walks, but are consistent over a longer timescale. The leader-follower network was hierarchical, and the dogs' positions in the network correlated with dominance order derived from everyday life situations. Leadership also correlated with age and personality traits such as trainability and aggression.

Our findings on the connection between variables extracted from GPS trajectory data, dominance rank, and personality traits could pave the way for automated animal personality and dominance measurements. As dogs are ideal models of human social behaviour [48], [49] and social robots [50], the present study may also be applied to measure social interactions in humans, as in the case of parents walking with their children, or humans interacting with robots.

## Materials and Methods

### Ethics statement

Non-invasive studies on dogs are currently allowed to be done without any special permission in Hungary by the University Institutional Animal Care and Use Committee (UIACUC, Eötvös Loránd University, Hungary). The currently operating Hungarian law “1998. évi XXVIII. Törvény” – the Animal Protection Act – defines experiments on animals in the 9th point of its 3rd paragraph (3. §/9.). According to the corresponding definition by law, our non-invasive observational study is not considered as an animal experiment. The owners volunteered to participate and gave written consent to the publication of the photos.

### Subjects

6 dogs (5 Hungarian Vizslas and one mixed breed; labelled V1 to V5 and M, respectively) and their owner took part in the experiments. Demographic characteristics are shown in Table 2. Photos of the subjects are presented in Figure S2, kinship is depicted in Figure S3.

### Procedure

GPS data were collected during 14 daily walking tours, each lasting about 30–40 minutes between 2 May 2010 and 25 November 2010. We analysed 823,148 data points. The high-resolution GPS devices were attached to the dogs with ordinary harnesses (Figures S1, S2), while the owner carried one device attached to her shoulder. The 5 Hz custom-designed GPS devices had a time resolution of 0.2 s and previous independent tests with the same devices showed a spatial accuracy of 1–2 m ([4] – Text S1). Weighing only 16 g, and with dimensions of 2.5 cm×4.5 cm, it is reasonable to suppose that the devices did not hinder the dogs' movements.

The group always walked on the same open grassy field, with the approximate dimensions of 500×1000 m, near Budapest, Hungary (located 47°25′17″N latitude, 19°8′45″E longitude).

The task of the owner was walk continuously and with a constant speed as far as possible during the walks. The dogs were allowed to walk and run freely, and the owner called the dogs back to herself only when she noticed some kind of danger, which happened on just a few occasions. Graphical summary of the Procedure is presented in Figure S1.

### Questionnaire surveys

The personality of the dogs was quantified using two questionnaires that were completed by the owner at the end of the GPS measurements.

1. The Dog Personality Questionnaire (DPQ) ([51]). DPQ was compiled from 1,200 descriptions culled from dog-personality literature, shelter assessments, and dog experts' input. A narrowed list was administered to more than 6,000 participants. Items were evaluated in terms of factor- and facet-loadings, content validity, internal consistency, inter-rater reliability, test-retest reliability, and predictive validity. Convergent criteria favoured five factors, labelled as Fearfulness, Aggression towards People, Activity/Excitability, Responsiveness to Training and Aggression towards Animals. Narrower facets within each factor were also identified. The DPQ has a 75-item and a 45-item form, but we used the latter one (Table 2).
2. The dominance questionnaire [29], to our knowledge, is the only questionnaire available, which was developed with the aim of assessing dominance. The questionnaire quantifies agonistic interactions between pairs of dogs. The owner had to answer four questions concerning each dog pairs: usually which one barks first when a stranger comes to the house (in a competitive situation, dominant dogs bark more [22], which dog licks the other's mouth more often (a submissive display, [52]), which one eats first when they get food at the same time and at the same spot (dominant animals have priority access to food, [4]), and which one wins fights (dominant animals are consistent winners, [4]). Dogs could receive 1 point for each question, and we summed up the points of each dog (Figure 2C, Table 2).

### Data analysis

To extract information concerning the interactions between group members, we used a directional correlation analysis [26] with a time window to quantify the fast, joint direction changes of pairs. Highly correlated direction changes of pairs are usually found only when two dogs interact by running a part of a loop together. The timescale of the owner's direction changes was much larger than that of the dogs, and – due to the short time window and the typically small time delays – it was not covered in the calculations. Therefore interactions between the owner and the dogs were not detected with this method. However, we know that the owner was walking on a predetermined route, and clearly led the whole group on a longer time scale (Figure 1, Figure S5 and Video S1).

We calculated directional correlation values for all short trajectory segments that were in a 6 s time window (t_win_; in other details the method was identical to [26]), thus isolating short-term effects. We used t_win_ = 6 s in the study, but the exact choice for the time window size has no substantial effect on the results (Figure S8). A local interaction event was defined to exist when corresponding trajectory segments had a higher correlation value than *C_min_* = 0.95 (Figure S7).

To extract leading tendency differences between members of pairs, the temporal directional correlation delay times (*τ_ij_*) were determined with the maximal correlation value. Positive *τ_ij_* values correspond to leading events when dog *i* leads dog *j*, as the direction of motion of *i* is ‘copied’ by *j* delayed in time. For each pair, leading-following events corresponding to different *τ_ij_* time delays were summed for each case in a walk, and for all 14 walks measured. For a detailed description of the applied method and a histogram of the found time delays between dog *i* and dog *j*, see Figure 2A and Figure S8.

If a clear maximum of the time delay histogram exists, it indicates frequent interaction between a dog pair at and near a well-defined time delay (see detailed description in Text S1 and Figures S8, S9). In many cases it can be seen from the histograms of those dog pairs where interaction was found (Figure 2A shows a typical example) that the leading and following roles (i.e. the sign of the time delay) are dynamically changing during a walk and also between walks. Significant deviation from zero in the location of the maximum value indicates that the dogs in the current pair have different leading propensities, suggesting a directed leader-follower interaction. The full width at half maximum of the histogram (see Text S1) characterises how stable the leader-follower relationship between a pair is.

We constructed an interaction network based on the detected interactions and leading tendency differences (Figure 2B, see also Figure S10). An edge (or link) indicates detected interaction between a dog pair. In those pairs where there is a significant difference in leading tendency we defined a directed edge (pointing from the dog who was found to lead more frequently to the one who more often assumes the role of follower).

The result of the method using the directed edges of the leadership network to characterise active connections was confirmed in an independent way. From the positional data we determined whether members of a pair spend more time in the close vicinity of each other compared to a randomized case (for more details see Text S1). This vicinity method does not require synchronised movement from interacting pairs. The resulting “social” network of the directional correlation and the vicinity method are in high correlation (two-tailed Pearson correlation, *r* = 0.600, *n* = 15 (number of possible pairs), *p* = 0.018).

## Supporting Information

Figure S1 **Graphical abstract of the study.**
(TIF)

Figure S2 **The owner and her dogs participating in the study.** Dogs wore a harness equipped with a GPS and moved freely during the walks.
(JPG)

Figure S3 **Genealogy of the Vizslas.** The colouring and shape of symbols indicate the sex of the individuals: yellow rounded boxes are females, blue rectangular boxes are males. The graph shows all relevant relationships between the subjects and their parents/offsprings.
(TIF)

Figure S4 **Illustration of the smoothing of the GPS trajectories, and its effect on the velocities calculated by numerical derivation.** For a 50 s long part of a track (dog V1 and walk 5), components of the positions and the velocities are shown: (**A**) *x* (blue) and *y* (green), (**B**) *v_x_*, and (**C**) *v_y_*. Red dashed curves show the data for the smoothed trajectories. (**D**) For a 10 s trajectory segment (indicated by a black dashed line on the left side panels), positions and velocities are shown. The velocities are depicted by vectors and are shifted to the right for better visibility.
(TIF)

Figure S5 **Illustration of the returns to the owner for dog V1 during the same walk that is presented on ****Figure 1****.** The parts highlighted with thick lines show the path travelled by the dog (red) and the owner (black) when our algorithm found the dog to be returning. Arrows indicate the distance between the dog and the owner at the beginning of the return (orange) and at the end (grey).
(TIF)

Figure S6 **Velocities of the dogs during walks.** Gray histograms show the speed PDFs of two dogs (V1 and V3; on Panel B, D and A, C, respectively), for two different walks. The curve on each graph shows the sum of the two lognormal functions which were fitted to the data. Two separate maxima are visible on each graph, the first represents time spent not moving (standing, digging, etc.), while the second indicates the preferred running speed.
(TIF)

Figure S7 **The distance and correlation-distance histogram of dogs for the cases when interactions were found by the time windowed directional correlation delay method.** (**A**) Histogram illustrating the frequency distribution of distances (bin = 2 m) for all pairs and walks summed up. (**B**) Histogram illustrating the frequency distribution of distances (bin = 2 m) and corresponding correlation values (C_ij_; bin = 0.001). Note that C_ij_ is related to the average difference between the direction of movement of the two dogs in a pair with the time delay providing the highest correlation: it gives the cosine of the angle between the directions (C_ij_ = 0.95 corresponds with 18.2°, C_ij_ = 0.99 with 8.1°). There was no need to use a cut-off limit for the distances, as most interactions occurred when the dogs were in the range of vision of each other. The C_ij_>0.95 criterion is sufficiently lax, as most detected interactions had much higher correlation values.
(TIF)

Figure S8 **Directional correlation delay time (τ) values for all possible pairings.** On each panel the grey histogram shows the frequency of the interactions detected with different time delays, when high correlation was found for a 6 s long time window (normalized with the number of walks). The curves show the functions gained by Gaussian smoothing with σ = 0.3 s for three different time window sizes: 4 s, 6 s and 8 s. For a shorter time window, more interaction events are found (the values are higher and lower for t_win_ = 4 s and 8 s, respectively). We used t_win_ = 6 s in the study, but the overall shape of the histogram remains unchanged, therefore the exact choice of 6 s for the time window size has no substantial effect on the results. The green histograms show the probability density functions of the bootstrapped sample histogram maxima, with the corresponding vertical axis on the right. The panels are arranged in ascending order of the S. D. of the bootstrapped maxima. This value was used to distinguish between the existence or absence of a significant peak. (**A–H**) Pairs where significant leader-follower relationships were found are shown with blue. The black dashed curves indicate Gaussian distributions fitted to the [−1 s; 1 s] range around the maximum of the given histogram, for the 6 s long time window. These Gaussian distributions were used to estimate the ratio of leading for each pair. (**I–O**) Those pairs where no significant connections were found in the absence of a significant peak are shown with red. See details of the decision criteria in Figure S9, and for the effect of this choice on the leadership network, consult Figure S10.
(TIF)

Figure S9 **Randomisation method for deciding when a histogram does or doesn't have a peak.** The black curve shows the cumulative distribution of the S.D. of bootstrapped maxima, for 4000 randomised histograms. We gained the randomised histograms by summing up the directional correlation delay time histograms of randomly selected pairs for each walk. The graph also shows the measured S.D. of the bootstrapped histogram maxima for every pair. Pairs where we detected significant leader-follower relationships are indicated with blue colour, otherwise red colour was used.
(TIF)

Figure S10 **The effect of the cut-off value for considering histograms to have a significant peak on the leadership network.** On the top, the maximal value of the S.D. of the bootstrapped maxima for accepting an interaction is shown. Lower or higher limits result in less or more edges in the network, respectively. However, the overall hierarchy remains the same. The numbers next to each node indicate the number of individuals which can be reached via directed links. This value was used as a measure of the leadership rank. The leadership network shown for lower (**A–B**) and higher (**D–E**) thresholds than the limit chosen (**C**) for use in the main text (Figure 2) and in all further analysis. At the bottom, for each network the Pearson correlation coefficient and the corresponding p-value is shown for the correlation between the leadership ranks, and the dominance ranks (based on [29], for the Vizslas (*n* = 5). In all cases the correlation is significant.
(TIF)

Figure S11 **Pearson correlation values between all variables extracted from the trajectory data, and the personality traits of the dogs (measured by questionnaires).** Cells that contain correlations with *p*<0.05 are in bold. Correlation values are colour-coded according to the corresponding p-values for positive correlation (blue: *p*<0.01; cyan: 0.01<*p*<0.05) and for negative correlation (green: *p*<0.05). The p-values are shown on Figure S12. An “x” indicates cells where correlation calculation is not applicable. Note that the correlations were determined using a small sample size of Vizslas (*n* = 5), therefore none of the p-values remain significant when correcting against multiple comparisons (Bonferroni, Sidak or Benjamini–Hochberg procedure), because of the large number of possible pairings (*n* = 300). Figure 3. presents the significant correlations in a network format.
(TIF)

Figure S12 **P-values between all variables presented on Figure S11.** Cells that contain correlations with *p*<0.05 are in bold. The values are colour-coded for positive correlation (blue: *p*<0.01; cyan: 0.01<*p*<0.05) and for negative correlation (green: *p*<0.05). An “x” indicates cells where correlation calculation is not applicable. Please check the details at Figure S11.
(TIF)

Figure S13 **Results of the permutation test performed to check the validity of the correlations shown on Figure S11.** For each variable pair, the Pearson correlation values were calculated for all possible permutations of the five Vizslas. The cells show the ratio of correlation values in the permuted cases that are higher than or equal to the correlation value of the correct pairing. Cells are highlighted with blue for positive correlations, where this ratio is below 0.025, and with green for negative correlations, where the ratio is above 0.975. An “x” indicates cells where correlation calculation is not applicable. Please check the details at Figure S11.
(TIF)

Table S1 **The variables characterising the interactions between pairs of dogs detected via the time-windowed directional correlation function method and the bootstrap method.** Note that where τ* is positive, the dog in the first column leads more often than the dog in the second column, and vice versa.
(DOC)

Text S1 **Supplementary details of the analysis, additional results, and justifications.** The supplementary text contains technical details of the data filtering and processing, justification of the variables by showing their uniqueness and consistency, justification of the correlations by an additional permutation test, and justification of all chosen parameters by showing that they have no effect on the final results.
(DOC)

Video S1 **Animation showing a 3 minute long part of a walk by the owner (black triangle) and her dogs (coloured circles), recorded with GPS devices.** In the bottom right corner the real time is shown, the video is played at 5 times the real speed. The inset in the top right corner illustrates the total path of the owner during the walk which started at the origin. The small rectangle shows the area presented on the main plot. On the main plot, for each individual the thick, normal and thin lines show the trajectories of the last 2 s, 5 s and 20 s, respectively. The momentary leader-follower relationships found by the time windowed directional correlation delay method are shown with the kite-shaped highlighting: between the smaller equal-length sides (close to the right angle vertex) is the leader, while the acute angle vertex points towards the follower.
(AVI)
