# Supplementary figures and images for: Leadership and Path Characteristics during Walks Are Linked to Dominance Order and Individual Traits in Dogs
Source: PLoS Comput Biol. 2014 Jan 23;10(1):e1003446. doi: 10.1371/journal.pcbi.1003446 (PMC3900374; doi:10.1371/journal.pcbi.1003446)

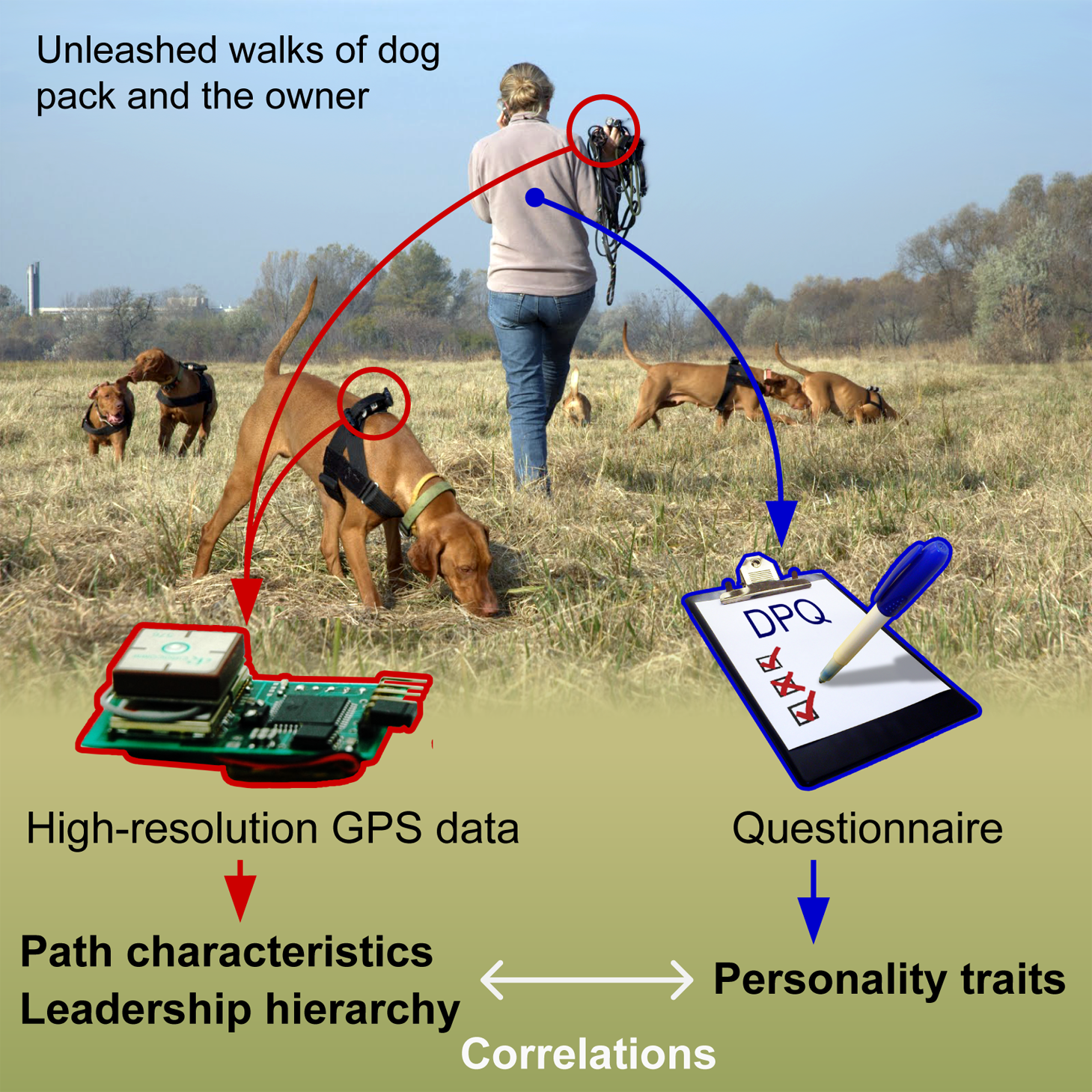

Supplement: Figure S1 — Graphical abstract of the study. (TIF) [file pcbi.1003446.s001.tif]

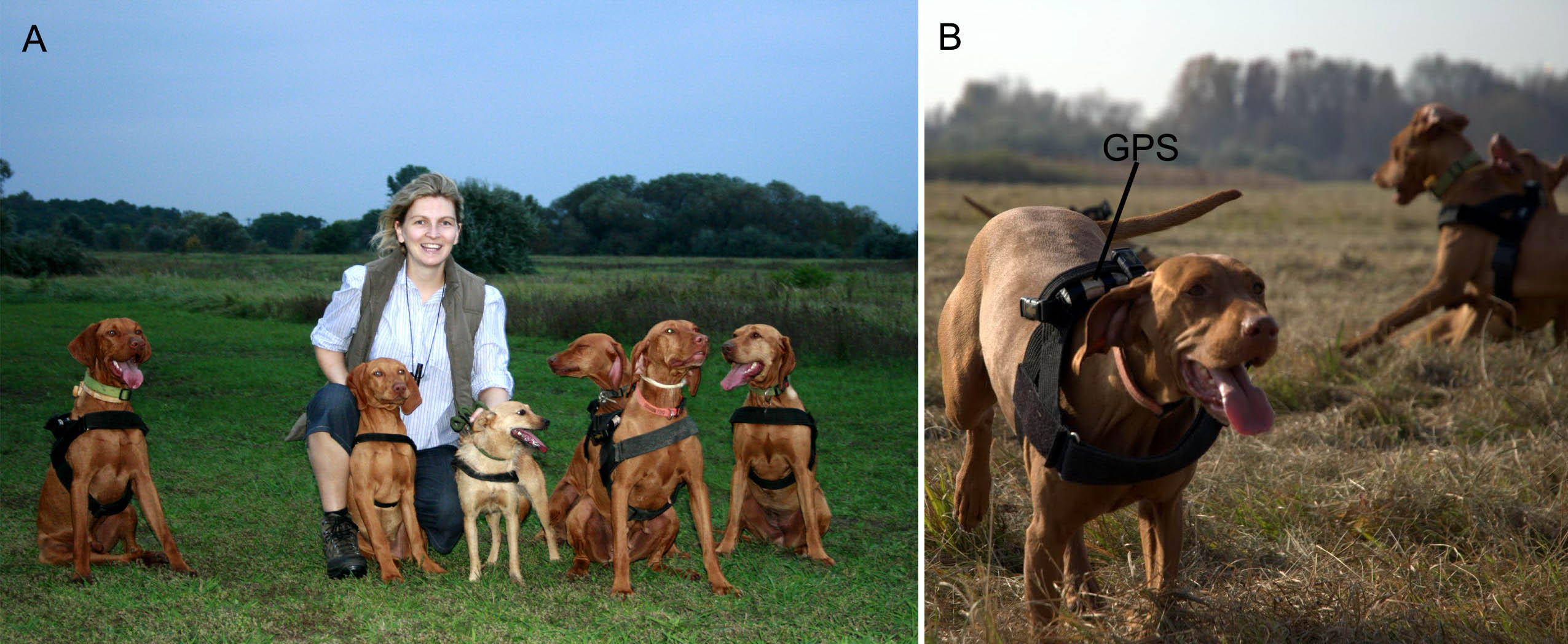

Supplement: Figure S2 — The owner and her dogs participating in the study. Dogs wore a harness equipped with a GPS and moved freely during the walks. (JPG) [file pcbi.1003446.s002.jpg]

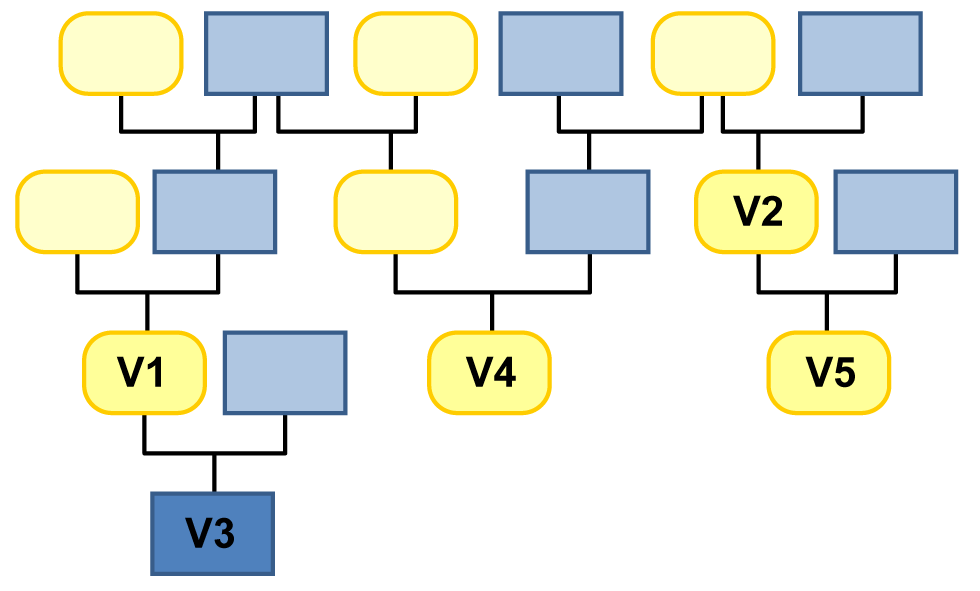

Supplement: Figure S3 — Genealogy of the Vizslas. The colouring and shape of symbols indicate the sex of the individuals: yellow rounded boxes are females, blue rectangular boxes are males. The graph shows all relevant relationships between the subjects and their parents/offsprings. (TIF) [file pcbi.1003446.s003.tif]

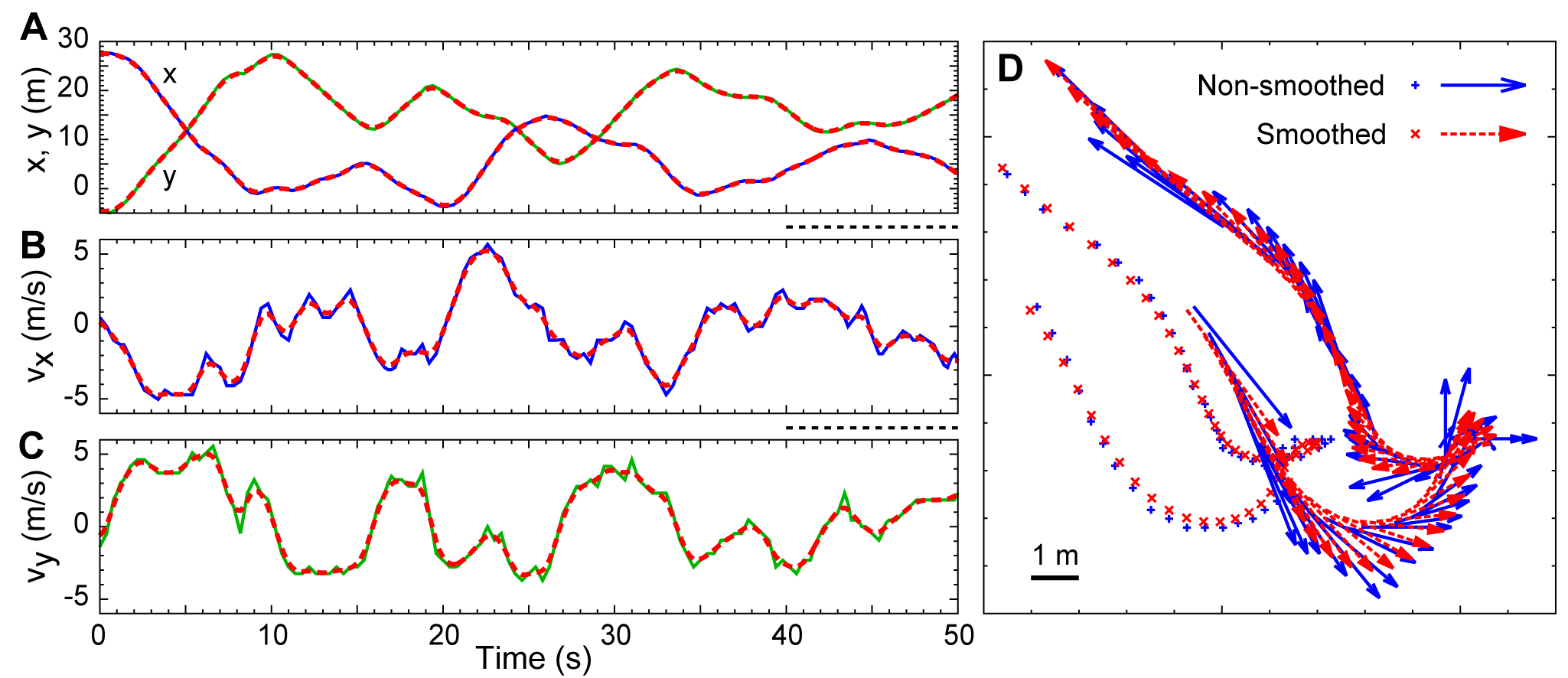

Supplement: Figure S4 — Illustration of the smoothing of the GPS trajectories, and its effect on the velocities calculated by numerical derivation. For a 50 s long part of a track (dog V1 and walk 5), components of the positions and the velocities are shown: (A) x (blue) and y (green), (B) vx, and (C) vy. Red dashed curves show the data for the smoothed trajectories. (D) For a 10 s trajectory segment (indicated by a black dashed line on the left side panels), positions and velocities are shown. The velocities are depicted by vectors and are shifted to the right for better visibility. (TIF) [file pcbi.1003446.s004.tif]

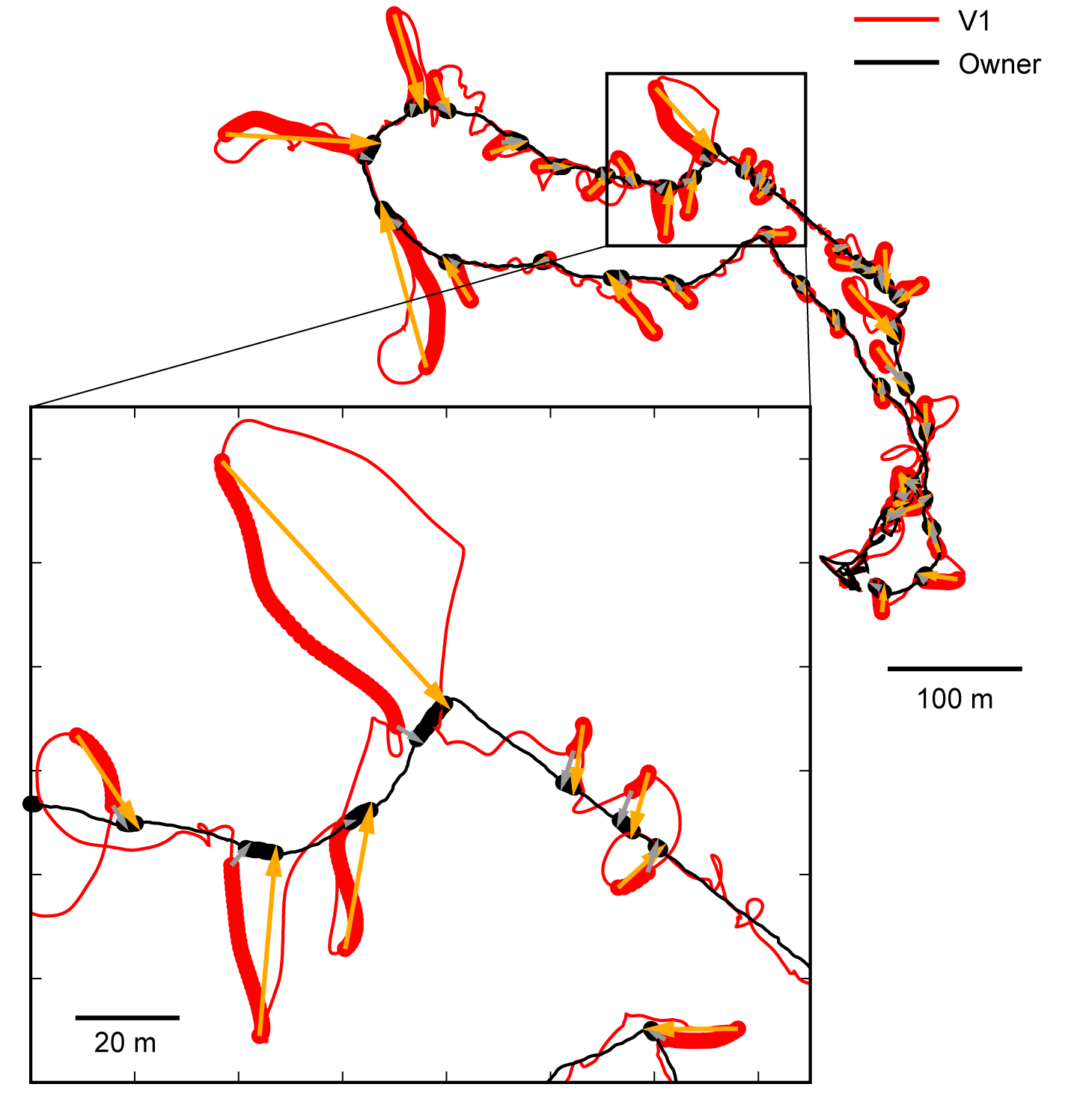

Supplement: Figure S5 — Illustration of the returns to the owner for dog V1 during the same walk that is presented on Figure 1. The parts highlighted with thick lines show the path travelled by the dog (red) and the owner (black) when our algorithm found the dog to be returning. Arrows indicate the distance between the dog and the owner at the beginning of the return (orange) and at the end (grey). (TIF) [file pcbi.1003446.s005.tif]

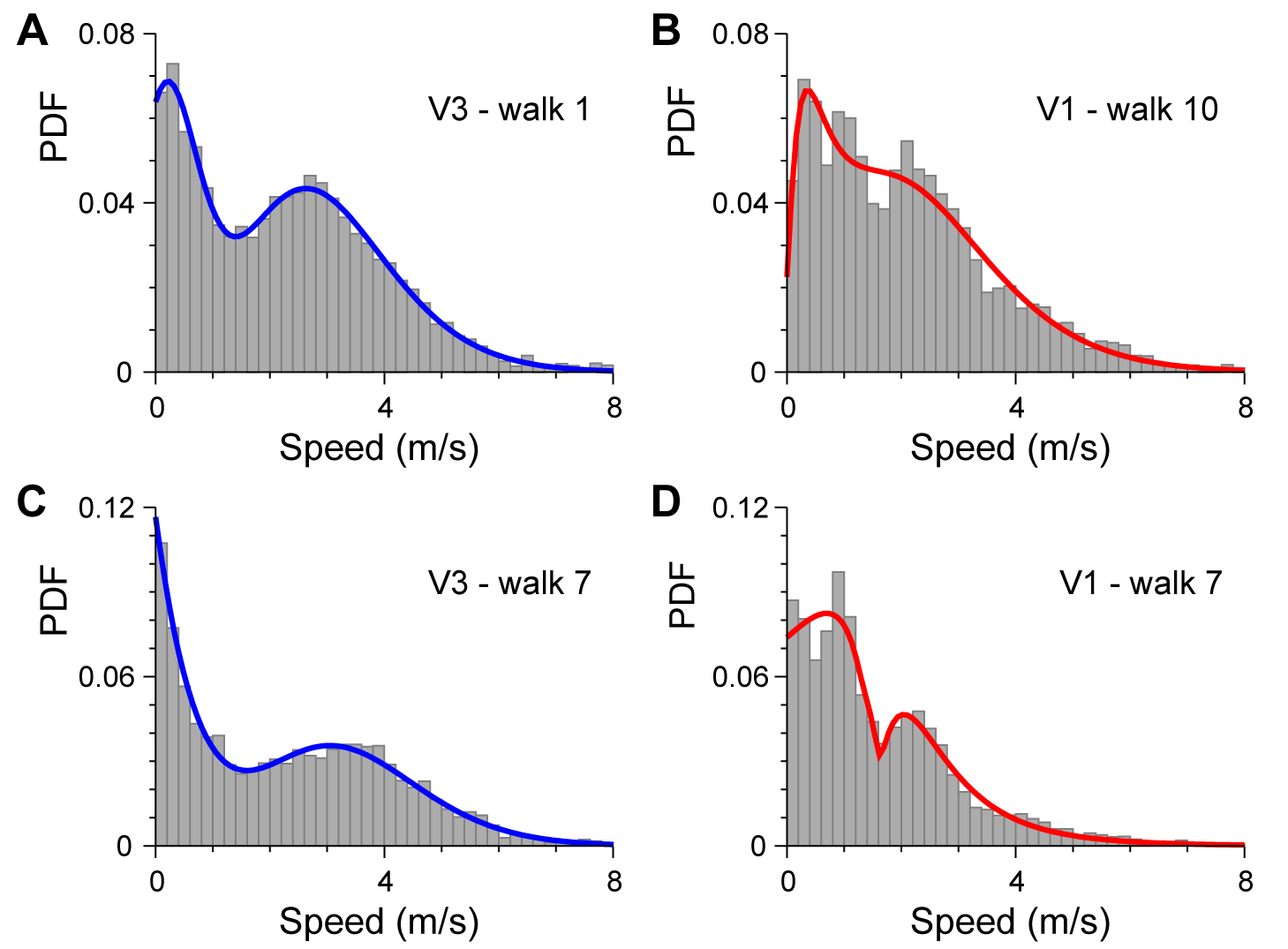

Supplement: Figure S6 — Velocities of the dogs during walks. Gray histograms show the speed PDFs of two dogs (V1 and V3; on Panel B, D and A, C, respectively), for two different walks. The curve on each graph shows the sum of the two lognormal functions which were fitted to the data. Two separate maxima are visible on each graph, the first represents time spent not moving (standing, digging, etc.), while the second indicates the preferred running speed. (TIF) [file pcbi.1003446.s006.tif]

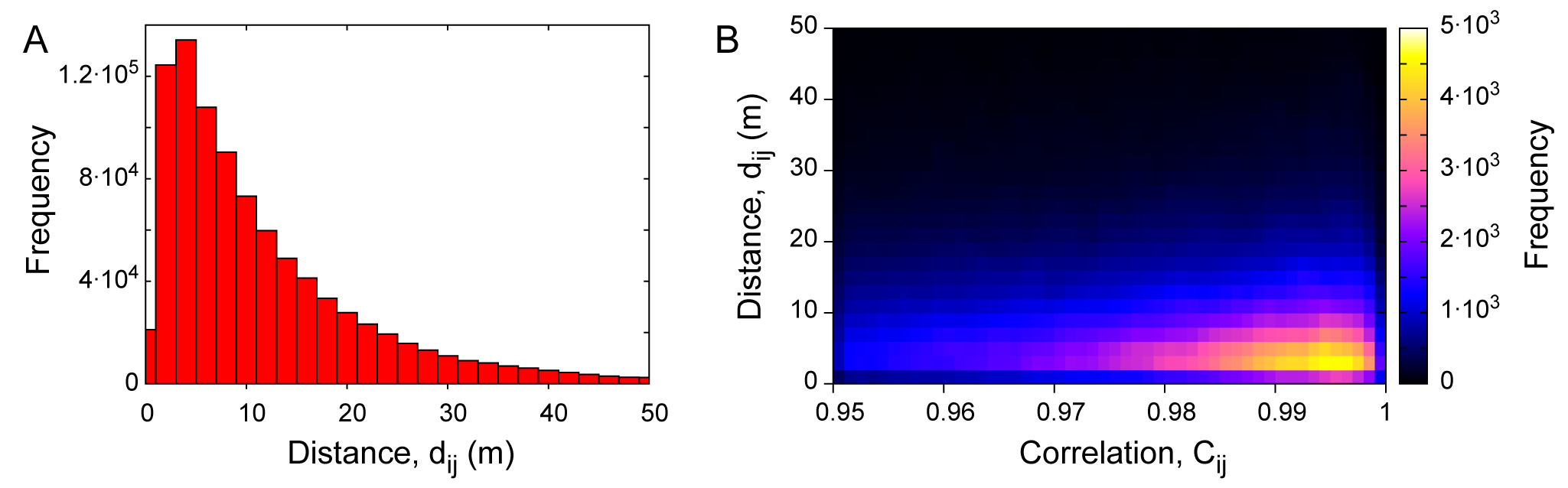

Supplement: Figure S7 — The distance and correlation-distance histogram of dogs for the cases when interactions were found by the time windowed directional correlation delay method. (A) Histogram illustrating the frequency distribution of distances (bin = 2 m) for all pairs and walks summed up. (B) Histogram illustrating the frequency distribution of distances (bin = 2 m) and corresponding correlation values (Cij; bin = 0.001). Note that Cij is related to the average difference between the direction of movement of the two dogs in a pair with the time delay providing the highest correlation: it gives the cosine of the angle between the directions (Cij = 0.95 corresponds with 18.2°, Cij = 0.99 with 8.1°). There was no need to use a cut-off limit for the distances, as most interactions occurred when the dogs were in the range of vision of each other. The Cij>0.95 criterion is sufficiently lax, as most detected interactions had much higher correlation values. (TIF) [file pcbi.1003446.s007.tif]

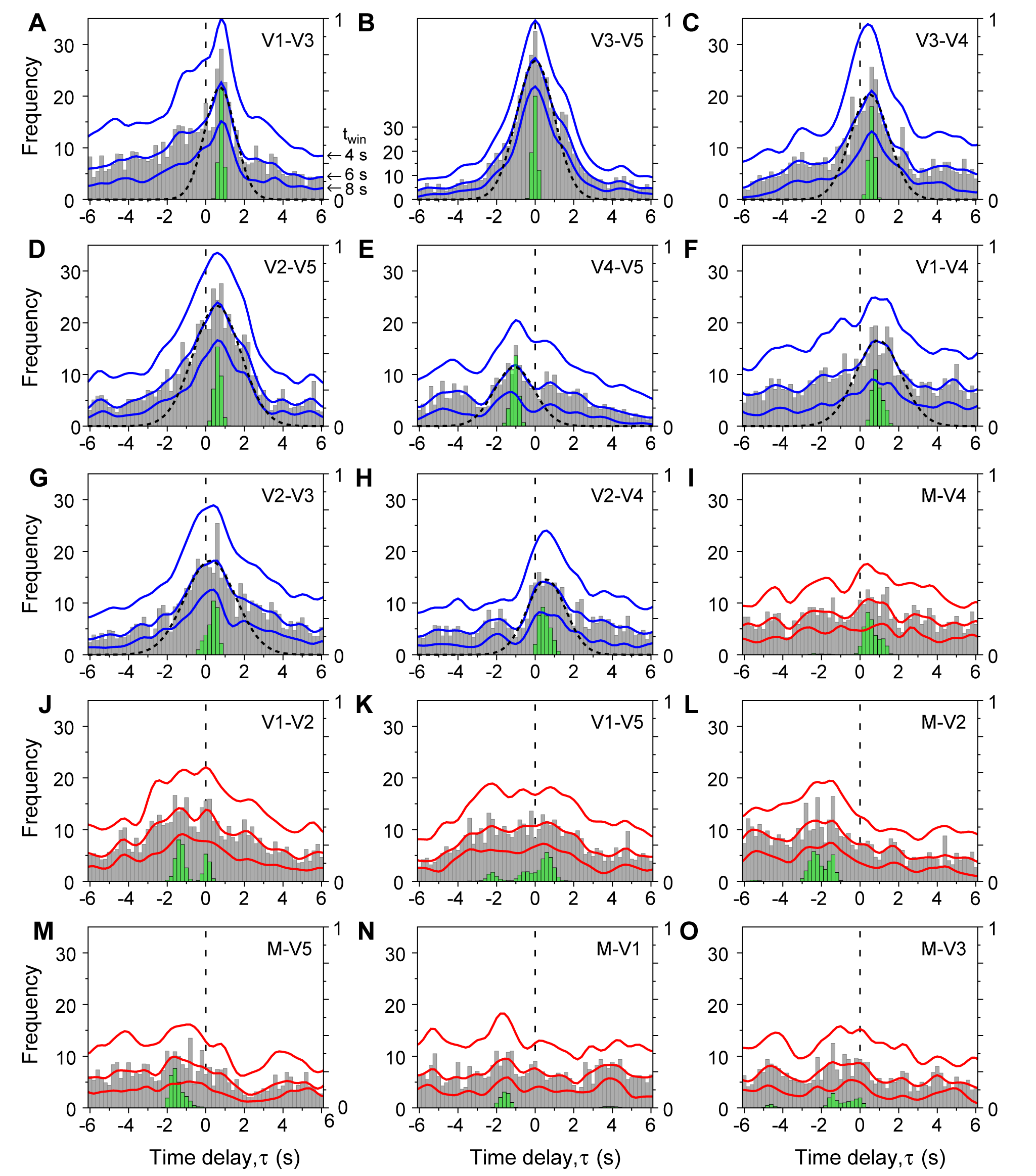

Supplement: Figure S8 — Directional correlation delay time (τ) values for all possible pairings. On each panel the grey histogram shows the frequency of the interactions detected with different time delays, when high correlation was found for a 6 s long time window (normalized with the number of walks). The curves show the functions gained by Gaussian smoothing with σ = 0.3 s for three different time window sizes: 4 s, 6 s and 8 s. For a shorter time window, more interaction events are found (the values are higher and lower for twin = 4 s and 8 s, respectively). We used twin = 6 s in the study, but the overall shape of the histogram remains unchanged, therefore the exact choice of 6 s for the time window size has no substantial effect on the results. The green histograms show the probability density functions of the bootstrapped sample histogram maxima, with the corresponding vertical axis on the right. The panels are arranged in ascending order of the S. D. of the bootstrapped maxima. This value was used to distinguish between the existence or absence of a significant peak. (A–H) Pairs where significant leader-follower relationships were found are shown with blue. The black dashed curves indicate Gaussian distributions fitted to the [−1 s; 1 s] range around the maximum of the given histogram, for the 6 s long time window. These Gaussian distributions were used to estimate the ratio of leading for each pair. (I–O) Those pairs where no significant connections were found in the absence of a significant peak are shown with red. See details of the decision criteria in Figure S9, and for the effect of this choice on the leadership network, consult Figure S10. (TIF) [file pcbi.1003446.s008.tif]

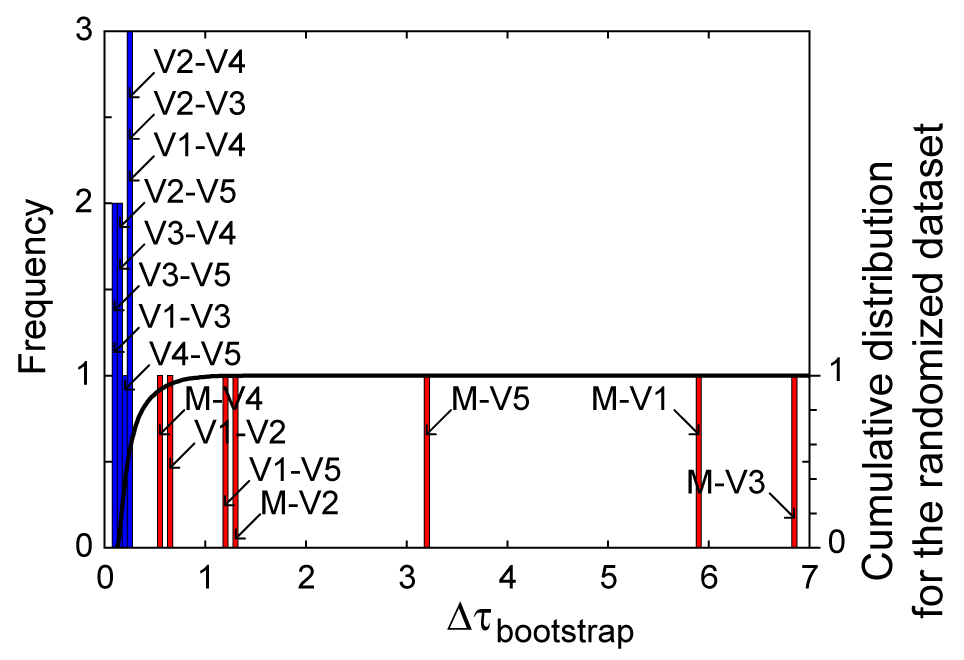

Supplement: Figure S9 — Randomisation method for deciding when a histogram does or doesn't have a peak. The black curve shows the cumulative distribution of the S.D. of bootstrapped maxima, for 4000 randomised histograms. We gained the randomised histograms by summing up the directional correlation delay time histograms of randomly selected pairs for each walk. The graph also shows the measured S.D. of the bootstrapped histogram maxima for every pair. Pairs where we detected significant leader-follower relationships are indicated with blue colour, otherwise red colour was used. (TIF) [file pcbi.1003446.s009.tif]

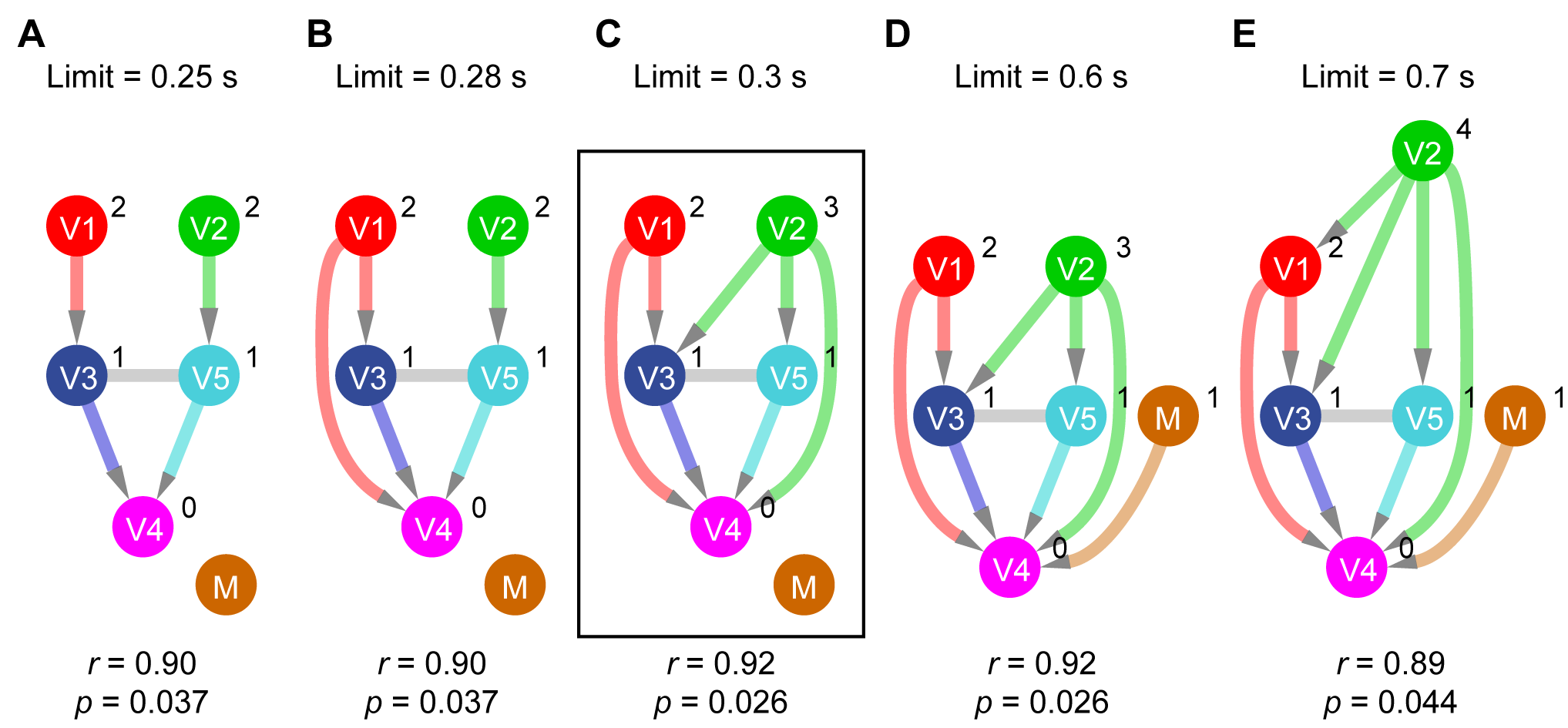

Supplement: Figure S10 — The effect of the cut-off value for considering histograms to have a significant peak on the leadership network. On the top, the maximal value of the S.D. of the bootstrapped maxima for accepting an interaction is shown. Lower or higher limits result in less or more edges in the network, respectively. However, the overall hierarchy remains the same. The numbers next to each node indicate the number of individuals which can be reached via directed links. This value was used as a measure of the leadership rank. The leadership network shown for lower (A–B) and higher (D–E) thresholds than the limit chosen (C) for use in the main text (Figure 2) and in all further analysis. At the bottom, for each network the Pearson correlation coefficient and the corresponding p-value is shown for the correlation between the leadership ranks, and the dominance ranks (based on [29], for the Vizslas (n = 5). In all cases the correlation is significant. (TIF) [file pcbi.1003446.s010.tif]

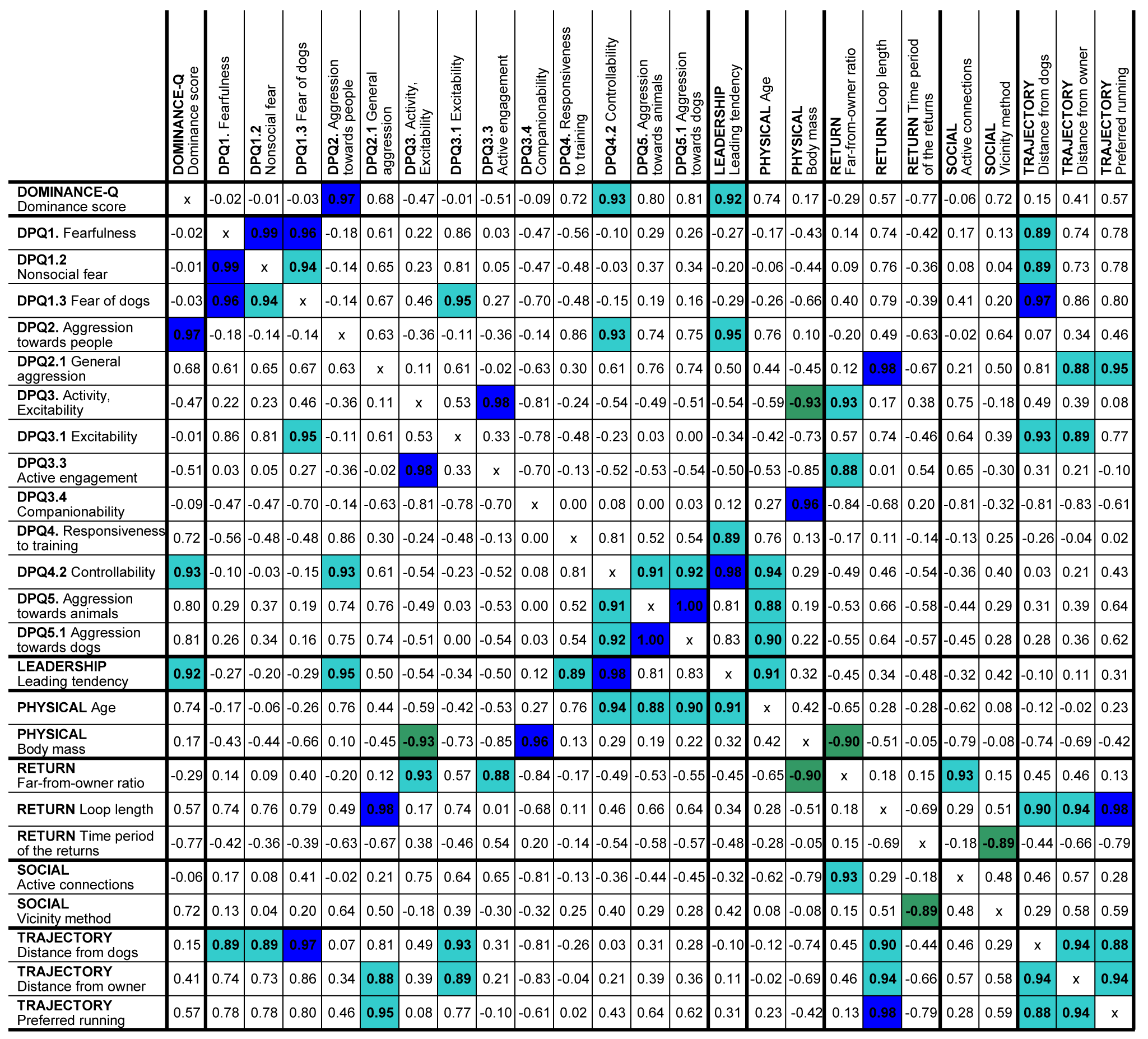

Supplement: Figure S11 — Pearson correlation values between all variables extracted from the trajectory data, and the personality traits of the dogs (measured by questionnaires). Cells that contain correlations with p<0.05 are in bold. Correlation values are colour-coded according to the corresponding p-values for positive correlation (blue: p<0.01; cyan: 0.01<p<0.05) and for negative correlation (green: p<0.05). The p-values are shown on Figure S12. An “x” indicates cells where correlation calculation is not applicable. Note that the correlations were determined using a small sample size of Vizslas (n = 5), therefore none of the p-values remain significant when correcting against multiple comparisons (Bonferroni, Sidak or Benjamini–Hochberg procedure), because of the large number of possible pairings (n = 300). Figure 3. presents the significant correlations in a network format. (TIF) [file pcbi.1003446.s011.tif]

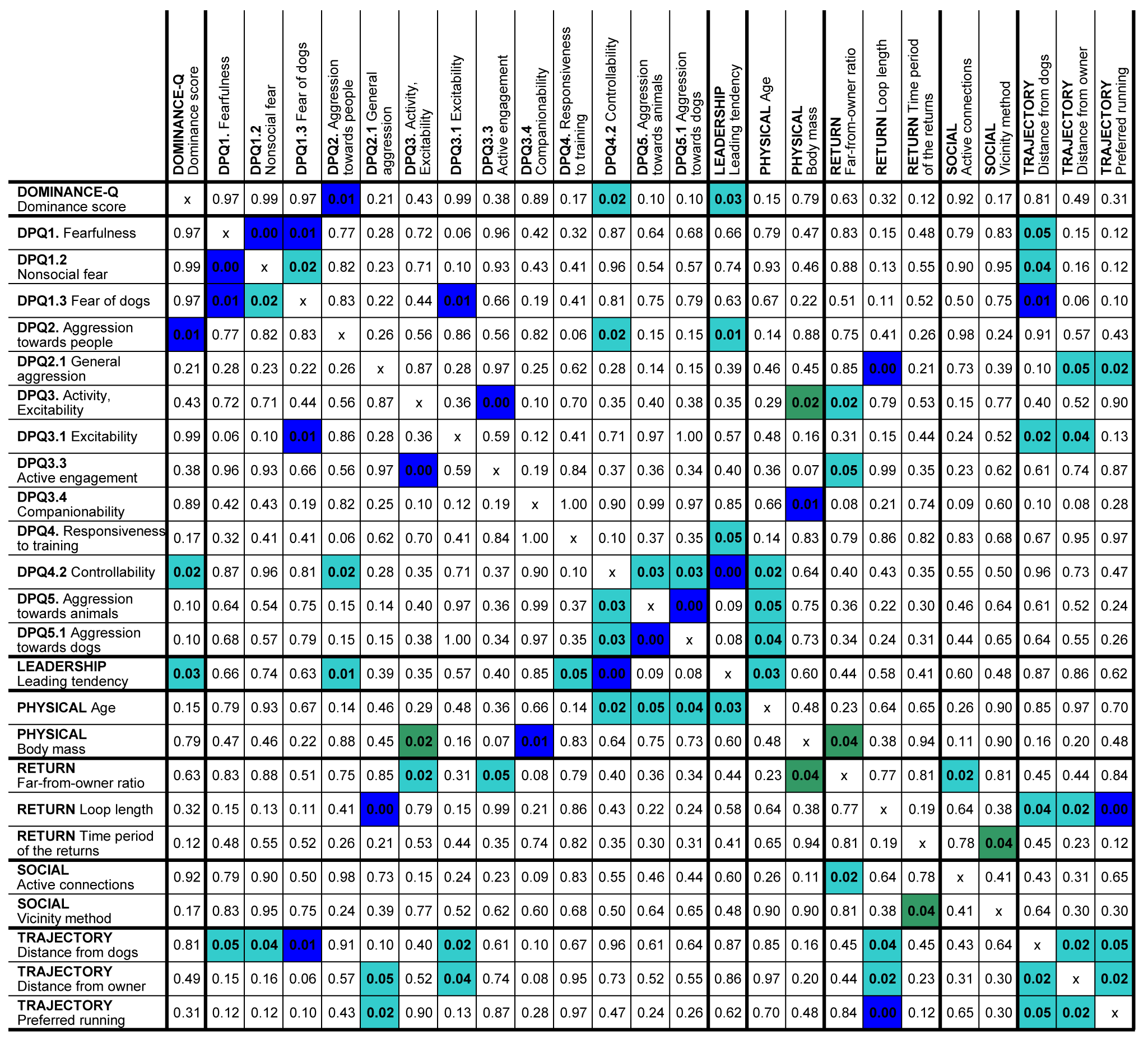

Supplement: Figure S12 — P-values between all variables presented on Figure S11. Cells that contain correlations with p<0.05 are in bold. The values are colour-coded for positive correlation (blue: p<0.01; cyan: 0.01<p<0.05) and for negative correlation (green: p<0.05). An “x” indicates cells where correlation calculation is not applicable. Please check the details at Figure S11. (TIF) [file pcbi.1003446.s012.tif]

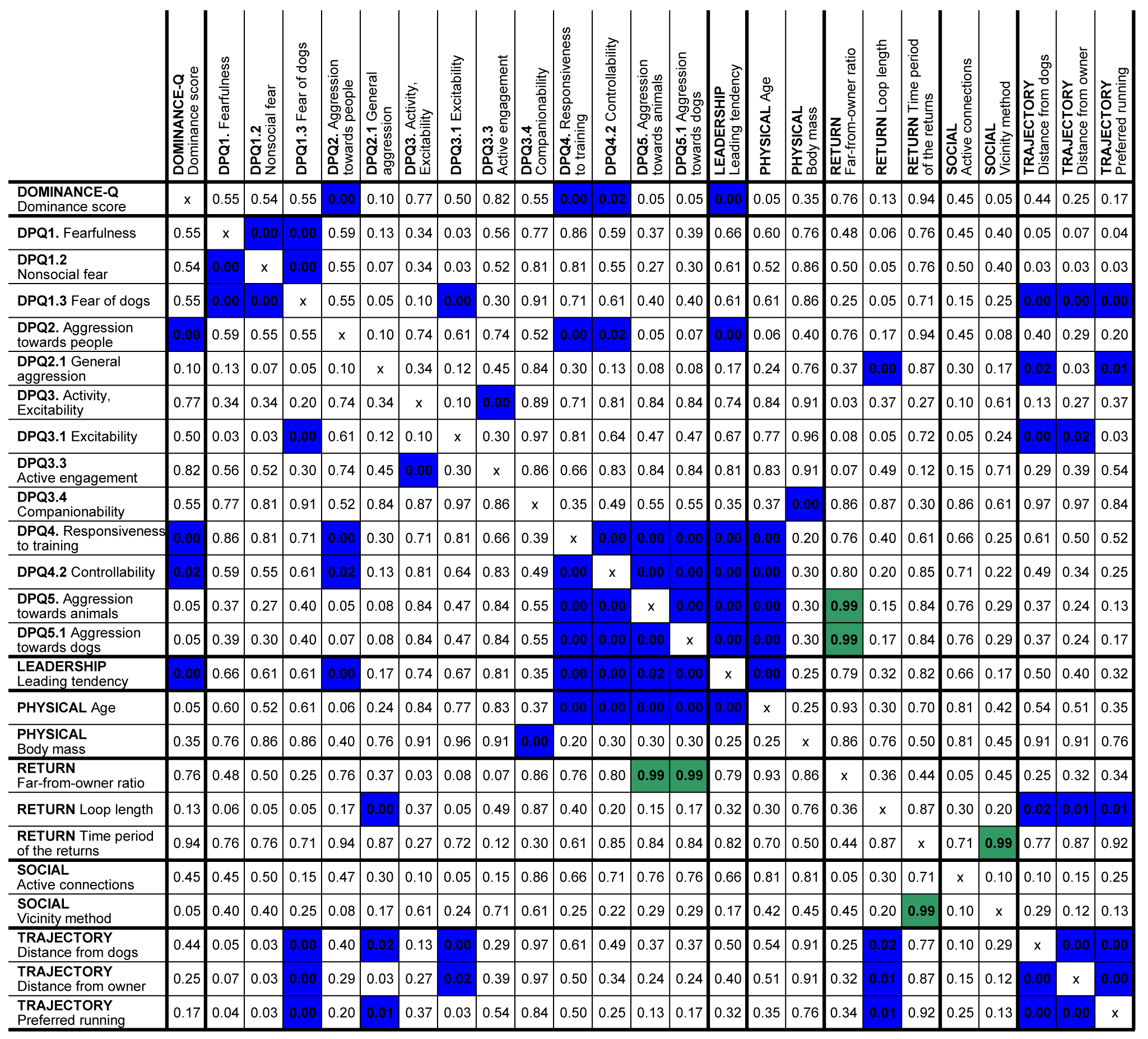

Supplement: Figure S13 — Results of the permutation test performed to check the validity of the correlations shown on Figure S11. For each variable pair, the Pearson correlation values were calculated for all possible permutations of the five Vizslas. The cells show the ratio of correlation values in the permuted cases that are higher than or equal to the correlation value of the correct pairing. Cells are highlighted with blue for positive correlations, where this ratio is below 0.025, and with green for negative correlations, where the ratio is above 0.975. An “x” indicates cells where correlation calculation is not applicable. Please check the details at Figure S11. (TIF) [file pcbi.1003446.s013.tif]
