# Supplementary material for: Leadership and Path Characteristics during Walks Are Linked to Dominance Order and Individual Traits in Dogs
Source: PLoS Comput Biol. 2014 Jan 23;10(1):e1003446. doi: 10.1371/journal.pcbi.1003446 (PMC3900374; doi:10.1371/journal.pcbi.1003446)
Supplement: Table S1 — The variables characterising the interactions between pairs of dogs detected via the time-windowed directional correlation function method and the bootstrap method. Note that where τ* is positive, the dog in the first column leads more often than the dog in the second column, and vice versa. (DOC) [file pcbi.1003446.s014.doc]

**Table S1. The variables characterising the interactions between pairs of dogs detected via the time-windowed directional correlation function method and the bootstrap method**. Note that where *τ** is positive, the dog in the first column leads more often than the dog in the second column, and vice versa.

| **Leader** | **Follower** | **Specific directional correlation delay time *τ** (s)** | **S.D. of the specific delay time Δ*τ** (s)** | ***τ** for the first 7 walks (s)** | ***τ** for the second 7 walks (s)** | **Full width at half maximum FWHM (s)** | **Ratio of leading** | ***τ** values of the non-smoothed tracks (s)** |
| --- | --- | --- | --- | --- | --- | --- | --- | --- |
| **V1** | **V3** | 0.8 | 0.13 | 0.5 | 0.6 | 3.4 | 0.82 | 0.8 |
| **V1** | **V4** | 0.9 | 0.27 | 0.6 | 0.8 | 5.2 | 0.79 | 0.9 |
| **V2** | **V3** | 0.3 | 0.29 | 0.3 | -0.5 | 4.2 | 0.57 | 0.4 |
| **V2** | **V4** | 0.5 | 0.29 | 0.3 | 0.4 | 3.2 | 0.72 | 0.6 |
| **V2** | **V5** | 0.6 | 0.18 | 0.5 | 0.2 | 3.6 | 0.68 | 0.6 |
| **V3** | **V4** | 0.6 | 0.16 | 0.1 | 0.4 | 2.6 | 0.69 | 0.6 |
| **V3** | **V5** | 0.0 | 0.13 | 0.1 | -0.3 | 2.8 | 0.50 | -0.2 |
| **V5** | **V4** | 1.1 | 0.20 | 1.2 | 0.5 | 4.8 | 0.85 | 1.4 |
