# Supplementary material for: Leadership and Path Characteristics during Walks Are Linked to Dominance Order and Individual Traits in Dogs
Source: PLoS Comput Biol. 2014 Jan 23;10(1):e1003446. doi: 10.1371/journal.pcbi.1003446 (PMC3900374; doi:10.1371/journal.pcbi.1003446)
Supplement: Text S1 — Supplementary details of the analysis, additional results, and justifications. The supplementary text contains technical details of the data filtering and processing, justification of the variables by showing their uniqueness and consistency, justification of the correlations by an additional permutation test, and justification of all chosen parameters by showing that they have no effect on the final results. (DOC) [file pcbi.1003446.s015.doc]

**Text S1**

**Supporting Information**

for

**Leadership and path characteristics during walks are linked to dominance order and individual traits in dogs**

by Zsuzsa Ákos, Róbert Beck, Máté Nagy, Tamás Vicsek and Enikő Kubinyi

# Preliminary data filtering

The GPS measurement data were first converted from geodetic coordinates into a Cartesian coordinate system and SI units by using the so-called Flat Earth Approximation and the WGS 84 system. The following equations describe the calculations made:

$R_{1}=\frac{R}{C}$, $R_{2}=\frac{R\left(1-f\left(2-f\right)\right)}{C^{3}}$,

$C=\sqrt{1-f\left(2-f\right)\sin^{2}\left(Lat_{0}\right)}$,

$R=6378137.0$, $f=\frac{1}{298.257223563}$,

$Lat_{0}=47^{\circ}25'37.27''$, $Lon_{0}=19^{\circ}7'59.87''$,

$x=\left(Lon-Lon_{0}\right)\cos\left(Lat_{0}\right)R_{1}$, $y=\left(Lat-Lat_{0}\right)R_{2}$,

where *Lat* and *Lon* are the measured geodetic latitude and longitude in radians, respectively, *Lat_0_* and *Lon_0_* are our chosen zero geodetic coordinates (used in radians), *R* is the radius of the Earth in meters, *f* is the flatness of the Earth, while *x* and *y* are the Cartesian coordinates in meters.

As the following step, the resulting trajectories were smoothed with a Gaussian filter (*σ* = 0.4 s) to reduce the measurement error of individual data points. The vertical (*Z*) coordinates had the highest measurement errors, and the movement took place on an effectively flat field; therefore, in all calculations only the horizontal (*X* and *Y*) coordinates were used. Velocity and acceleration values were gained by calculating numerical derivatives.

When the GPS devices temporarily lost their satellite connection, data points were missing from the trajectories. These points were filled in with a linear interpolation of nearby valid values. However, in all subsequent calculations, only those data points were used, which had at least 2 valid measured points in their ± 0.4 s neighbourhood, i.e. at least 2 out of 5 possible nearby points were recorded by the GPS.

The effects of measurement errors and missing data points on the calculations were tested in detail in a previous article for the same GPS devices, see SI of [26]. In order to check the effect of the Gaussian smoothing on the results of our correlation analysis, we ran the algorithms on the non-smoothed data, too. The *τ** specific directional correlation delay times of the detected interactions (see below, Directional correlation in time windows section) in the smoothed and non-smoothed cases proved to be significantly correlated (two-tailed Pearson, *r* = 0.97, *n* = 8, *p* < 0.0001), with only a few values changing slightly (values for the non-smoothed case are shown in the last column of Table S1). Therefore, apparently the smoothing introduces no distortion to the final results, but it reduces the noise of the velocities derived from the GPS position data (Figure S4).

Where we used not only the absolute value of the velocities, but their direction in our calculations, too, additional points had to be filtered out. When a subject stopped, the GPS measured points around its actual position with some error. After applying the Gaussian filter, the resulting velocities were of very small absolute value, but with an arbitrary direction. We only used those velocity direction values which had at least one data point farther than 3 m in their ± 3 s neighbourhoods. Thus, we required an average movement speed of 1 m/s nearby a data point to consider the dog to be moving at that time. This applied only to the dogs, as the owner was walking constantly.

The code for the conversion and filtering of the trajectory data is available on request.

# Characteristics of the paths

The **preferred** **running speed** was determined based on the maxima of the probability density function of speed values (see below, Preferred running speed section). The average of the preferred running speeds of different dogs over the different walks varied from 1.4 m/s (dog M, mixed-breed) to 4.0 m/s (dog V5, Vizsla), with significant differences between dogs (see below, Consistency and uniqueness of the measured variables section; also see Table 1).

The **relative distance covered** was calculated the following way: the actual distance covered by each dog was divided by the distance covered by the owner.

Also, we found significant differences in the relative distance covered by the dogs during the walks (see below). Taking into account the differences in the running speeds this is not surprising; however, it is not evident as dogs stopped and walked, too, during the walks. As the path length of the group varied during the different walks we provide the covered distance compared to the owner’s trajectory length. The slowest (mixed-breed) dog covered about 1.5 times the distance, while the fastest dog covered 3.7 times the distance that the owner did (the averages are 1.8 for Vizslas and 2.3 for adult Vizslas) (Table 1).

Average pair distances were calculated for every possible pair, including the owner. As different walks lasted for different time intervals, the final average pair distance in each case was calculated from the time weighted average pair distance values of the different walks. The **distance from the owner** was also found to be a unique and consistent feature of the dogs’ path (see below). The values varied from 9 m (dog V4, juvenile dog) to 23.3 m (dog V5) (Table 1). The **distance from dogs** (it is calculated as the average of the dog’s average pair distances with respect to all the other dogs, and it is a measure indicating the extent to which a dog stays in the vicinity of its group members) were varied from 16 m (dog V4, juvenile dog) to approximately 20 m (dog V5) (Table 1). The average distances from dogs variable was less distinctive between individuals (see below)

As we mentioned in the main body of the article, dogs prefer running in “loops” during the walks, which means they frequently run away from, and a short time later return to the owner. To investigate this behaviour we developed a method that automatically detects the returning events (for a detailed description, see the Returns to the owner section below). Figure S5 shows a typical trajectory of dog V1, indicating the returning events found with the algorithm.

The average **time period of the returns** to the owner (i.e. the average time between returning events) and the average **loop length** were determined for all dogs. To characterise to what extent a dog seeks the spatial vicinity of the owner during walks, we calculated the **far-from-owner ratio** (see below, Returns to the owner; also see Table 1). Each dog was faster than the owner, and their movement radius was not restricted during the walks, therefore they could hold any distance from the owner freely. In spite of this, we found that the dogs’ preferred running speed predicts very well the average length of the returning loops (*r* = 0.98, *n* = 5, *p* = 0.003) and the average distance from the owner during walks (*r* = 0.94, *n* = 5, *p* = 0.017; *r* = 0.85, *n* = 6, *p* = 0.032). In the analysis we used two-tailed Pearson correlation for all subjects (*n* = 6) and for the Vizslas only, too (*n* = 5) where normality of the variables were validated by Shapiro-Wilk test (P < 0.05) and Spearman correlation otherwise. In other words, faster, more agile dogs also run longer loops (they are able to run longer loops without lagging behind the owner) and, not coincidentally, their average distances from the owner are longer. All these features of the trajectories characterise the general “moving activity and ability” of the dogs during walks in a strong correlation with each other.

The described loop-like moving pattern of the dogs around the owner implies that the average distances between dog pairs can be affected by their running speed differences. As expected, if the dogs moved around the owner independently with a similar, loop-like moving pattern, slower dogs running shorter loops would have smaller average pair distances between each other. With respect to all the dogs, the average value of the average pair distances correlated with the dogs running speeds (*r* = 0.88, *n* = 5, *p* = 0.049), strengthening the expectation that running speed has an effect on pair distances. Because of the described effect it can be seen that simple assumptions on pair distances (e.g. that shorter distance would correspond to stronger interaction) cannot effectively characterise the observed dog pair interactions.

Average pair distance of the two fastest dogs (dog V5 and dog V3) was smaller than their respective average distances from all the other dogs. Based on the above discussed effect we would expect that the two fastest dogs would have the longest distance between each other if they would move independently. This indicates that there is some preference between dogs, and pair distances are not determined just by the dogs’ speeds.

To uncover social interactions during the walks we used two different methods that were more complex than the comparison of simple pair distances (see below).

# Preferred running speed

We calculated the probability density function (PDF) of the absolute value of the velocity for each dog, separately for all measurement runs. Typically, the speed PDFs had two distinct maxima. The first maximum corresponds to the time when dogs slow down, stand or dig (the speed values are never exactly 0 because of GPS measurement errors). The second maximum corresponds to the time spent running, thus it indicates a preferred running speed. To determine the maximum value, the sum of two lognormal curves was fitted to the values of the speed PDF. Figure S6 shows typical probability density functions of a dog’s speed values, along with the fitted curves. The average and standard deviation of the location of the second maximum – calculated for the different measurements – give the preferred running speed and its variability. The error of the fitted parameters was negligible compared to the differences seen between separate walks. The numerical results can be seen in Table 1.

# Returns to the owner

We developed an automated algorithm to detect the dogs’ frequent, periodic returns to the owner, as observed during the measurements. The definition of a returning event is the following: each continuous segment of a dog’s trajectory corresponds to a return if the angle between the dog’s velocity vector and the position vector pointing from the dog to the owner is constantly smaller than 45 degrees; the dog’s final distance from the owner is less than the half of the distance at the starting point of the returning event; and the distance was reduced by at least 3 m. These criteria, respectively, ensure that the dog is continuously moving in the general direction of the owner, that the distance to the owner is significantly reduced, and that actual separation is needed for an event to be recorded. The criteria are relatively lax, and their actual choice could be different without modifying the qualitative results, or the relations between the results for different dogs. Figure S5 shows a number of detected events; also see Figure 1.

After detecting the returning events, we calculated the time between consecutive ones. We call this measure time period of the returns to the owner (and we also calculated its reciprocal, the so-called return frequency). We calculated loop lengths (the length of the returning segments of the loops). The observed distributions of the time periods of the returns to the owner and the loop lengths showed heavy-tailed behaviour with a high probability for extreme values. As the number of detected returning events per walk for each dog was around 10-50, using the means of different walks would not have been meaningful. Therefore, we used the mean and S.D. of all detected events (during all walks) in order to characterise the behaviour of the dogs relative to each other. The results are shown in Table 1.

Moreover, we wished to differentiate between dogs that return rarely, but spend the majority of the time near the owner, and dogs that return rarely, but are most often far away from the owner, as these cases signal differing degrees of connection with the owner.

In order to do this, first we had to establish what counts as ‘close’in the case of each dog (as their mobility is different). We analysed the distributions of the distances from the owner at the starting and ending points of the returning events, and determined that about 15% of the data were outliers, as around 15% of the starting points were closer to the owner than 15% of the ending points. Therefore we decided that a dog can be considered close to the owner when it is closer than the 85th percentile of the distances at the ending points of its returns.

Thus, a dog could be in three states: returning, close to the owner, and far from the owner. The time ratio of being far from the owner (far-from-owner ratio) is expected to inversely characterise the strength of the dog’s connection to the owner. The data are shown in Table 1.

# Uniqueness and consistency of the measured variables

To investigate whether our measured variables were unique to individual dogs, we performed a one-way analysis of variance (ANOVA) tests for all dog pairings (checking whether the data of two dogs from different walks could be sampled from the same distribution).

The preferred running speeds, the relative distances covered, and the average distances from the owner all proved to be unique traits, with significant differences between dogs (one-way ANOVA, *p* < 0.05 for 12, 12 and 10 dog pairs, respectively, out of a total of 15 possible pairings).

In the case of the average distances from dogs and the far-from-owner ratios, the differences were less marked between individuals (one-way ANOVA, *p* < 0.05 for 3, and 3 pairs, respectively, out of 15).

In order to test the consistency of the measured variables during the 14 walks, we calculated the correlation between their average value for the first 7 and the last 7 walks. Positive correlation (two-tailed Pearson correlation, *n* = 6) were found for preferred running speed: *r* = 0.99, *p* < 0.001, relative distances covered: *r* = 0.96, *p* = 0.003, distance from the owner *r* = 0.96, *p* = 0.002, far-from-owner ratio: *r* = 0.93, *p* = 0.008. In the case of distance from dogs the correlation was not significant (*r* = 0.76, *p* = 0.079).

# Permutation test for the correlation between variables

Given the small sample size (for Vizslas *n* = 5), the p-values of Pearson correlations might need further justification. Therefore, to check the significance of the correlations between variables, we performed a permutation test. .

For all variable pairs, all possible permutations of the 5 individuals (119, excluding the correct one) were considered. We calculated Pearson correlation for all cases where the instances of the first variable were matched to the dogs in the correct order, while the instances of the second variable were matched to a permuted case.

We then created a cumulative distribution from the obtained correlation values, and determined whether the correlation value of the correct pairing was signifantly higher or lower than the randomised case, i.e. above the 0.975 or below the 0.025 thresholds of the cumulative histogram.

All correlations between variables mentioned as ‘significant’ in the article were found to have either significantly higher or lower correlation values than the randomised case, while at the same time satisfying the significance condition of the Pearson p-value.

# Directional correlation in time windows

We wished to quantify the interactions between dogs that ran similarly shaped loops together, as observed during the walks. We used directional correlation functions for this purpose, which were introduced in [2] to determine the leadership hierarchy of a pigeon flock. The directional correlation function is defined in the following way:

$$C_{ij}(\tau)=\left\langle\frac{\mathbf{v}_{i}(t)\cdot\mathbf{v}_{j}(t+\tau)}{\left|\mathbf{v}_{i}(t)\right|\cdot\left|\mathbf{v}_{j}(t+\tau)\right|}\right\rangle$$

Where **v***_i_*(*t*) is the normalised velocity vector of individual *i* at time *t*, while < > denotes time average. The τ value is a time delay that can characterise the temporal relationship between the direction changes of two subjects.

When a high correlation maximum is detected, for example, for a positive τ delay, it means that individual *j* frequently copies the movement direction of individual *i*, delayed by a specific amount of time. Thus, it can be said that individual *j* follows the movement of individual *i*, i.e. *i* leads *j*. If a clear maximum exists at a negative τ value, the inverse is true.

Contrary to pigeons [26], the interactions between pairs of dogs occur only in a relatively small ratio of the total time of the walks, when they run loops together. To isolate these short-term, but highly correlated effects, the time average of the directional correlation function was calculated for every 6 s long time window (one window for dog *i*, another window delayed by τ for dog *j*). A time window contains 30 data points, which is a compromise between locality and accuracy of the correlation values.

We selected those time windows that had a high correlation maximum of over C_min_ = 0.95 (see Figure S7), and recorded the *τ_ij_* directional correlation delay time values corresponding to the maximum. At least 90% of the data point pairs in the two windows had to be valid, and the dogs had to be moving at those points (see Preliminary data filtering).

The chosen length of the time window has no substantial effect on the final results. To show this, we plotted the directional correlation delay times found for each pair on Figure S8 using smaller (t_win_ = 4 s) and larger (t_win_ = 8 s) time windows.

It is important to note that directional correlation values are also very high for trajectory segments where, for example, both individuals go in the same direction in a straight line, therefore there is no similar direction change. However, in these cases a specific time delay value that is consistently detected over the walks does not exist. Thus, such events constitute the random noise in our measurements, while the similar direction changes generate the signal.

We summarised the detected *τ_ij_* directional correlation delay times for each pair of dogs. For single measurement runs, there were not enough detections to differentiate between the signal and the noise, so we summed up the histograms of *τ_ij_* values for all the walks. The resulting histograms were smoothed with a Gaussian filter of *σ* = 0.3 s.

**Method and criteria for choosing dog pairs with significant interactions**

For some dog pairs, a clear peak was apparent on the resulting smoothed histograms. We calculated the location of these empirical maxima, the specific directional correlation delay times (*τ**). To characterise the accuracy of these measures, we used a simple resampling-type bootstrap method [3]: we randomly sampled histograms (with replacement, using a Mersenne twister random number generator) from the original smoothed directional correlation delay time histograms, with the same total number of data points as the original ones. These bootstrap samples have similar properties to the original histogram, so we recorded the locations of their maxima, for 10000 samples per dog pair.

When we observed a clear peak on the original histogram, these bootstrapped maxima naturally showed a very narrow distribution, while for less substantial peaks, they came from a wider range of values. We calculated the average and the standard deviation of the bootstrapped maxima for each pair. We used the average value as the estimator of the ‘actual’ *τ** specific directional correlation delay time, while the Δ*τ** standard deviation characterises both how accurate the *τ** specific time is, and how well-determined a peak on a histogram is (with higher S.D. corresponding to lower peaks or no peak). Figure S8 depicts the original directional correlation delay time histograms along with the histograms of the bootstrapped maxima, while the numerical results are shown in Table S1.

To differentiate between clear, high peaks and inhomogeneities in the noise on the directional correlation delay time histograms, we used a randomisation method combined with the bootstrap method mentioned in the previous paragraph. We created randomised directional correlation delay time histograms by summing up the daily measured histograms of randomly selected dog pairs for 14 walks, then performed the bootstrapping analysis for 4000 of these histograms to get the standard deviation of their bootstrapped maxima. We compared the cumulative distribution of the randomised Δ*τ** values to the observed Δ*τ** values of the actual measured histograms (see Figure S9), and determined that histograms having Δ*τ** < 0.3 s should be considered to have a clear peak. In turn, a clear peak on a directional correlation delay time histogram corresponds to a consistent leader-follower interaction between a dog pair, with *τ** specific directional correlation delay time.

Note that the chosen limit of 0.3 s does influence the inclusion of single links in the network, but changing it does not affect the overall hierarchy (see Figure S10).

For the thus detected interactions between dog pairs, the sign of the *τ** specific directional correlation delay time indicates which dog assumes the role of the leader more frequently in their pairwise relationship. But these roles are dynamic, and often interchanged, as evidenced by the large full width at half maximum (FWHM) values of the histograms, shown in Table S1.

Using the calculated *τ** specific directional correlation delay times, we constructed the leadership network of the dogs, see Figure 2B. The variable leading tendency was determined for each dog using this network: it is defined as the ratio of the dogs that can be reached through edges pointing to followers, i.e. the number of direct and indirect followers summed up, divided by the number of dogs. Active connections is another simple network variable, it is the number of edges (connections) of each dog.

# Vicinity method

As we mentioned in the main body of the article, simple pair distances expectedly could also characterise which dogs seek each other’s companionship. However, we showed earlier that simple pair distances are affected by the moving activity of the dogs, therefore we applied the following method to determine whether a dog pair’s average pair distance is lower than what we would expect based exclusively on their moving activity.

The trajectory data were transformed to a co-moving coordinate system centred on the owner for every walk. This transformation allowed us to compare the average pair distances between trajectories of dogs selected from different walks, the owner being the reference point. Thus, by calculating the average distance between two dogs for all trajectory pairs coming from different measurement runs (the ‘randomized case’), we get a value containing the effects caused by the differing moving activities, but not those that are caused by actual interactions, consequently giving us a good reference value.

If the average distance between a dog pair (for the trajectories of the same measurements) is significantly lower than the reference value (75% of the reference value or lower), we can say that they seek each other’s proximity, and that we detected interactions between them. This method does not require joint fast movements from the pairs; therefore, it is possible that one of the dogs is passive in this interaction while the other one seeks its companionship.

The ‘social network’ constructed from the interactions detected with the vicinity method correlates positively with the network resulting from the time-windowed directional correlation analysis (two-tailed Pearson correlation, *r* = 0.600, *n* = 15 (number of possible pairs), *p* = 0.018).

The interaction number of a dog was determined as follows: the number of dogs with whom it has a significantly smaller average pair distance than the distance calculated in the randomized case. We call this variable vicinity method in our analysis.
